# IFN-γ is required for cytotoxic T cell-dependent cancer genome immunoediting

**DOI:** 10.1038/ncomms14607

**Published:** 2017-02-24

**Authors:** Kazuyoshi Takeda, Masafumi Nakayama, Yoshihiro Hayakawa, Yuko Kojima, Hiroaki Ikeda, Naoko Imai, Kouetsu Ogasawara, Ko Okumura, David M. Thomas, Mark J. Smyth

**Affiliations:** 1 Division of Cell Biology, Biomedical Research Center, Graduate School of Medicine, Juntendo University, Bunkyo-ku, Tokyo 113-8421, Japan; 2 Department of Biofunctional Micribiota, Graduate School of Medicine, Juntendo University, Bunkyo-ku, Tokyo 113-8421, Japan; 3 Department of Immunology, Juntendo University School of Medicine, Bunkyo-ku, Tokyo 113-8421, Japan; 4 Cancer Immunology Program, Peter MacCallum Cancer Centre, St Andrews Place, East Melbourne, 3002 Victoria, Australia; 5 Frontier Research Institute for Interdisciplinary Sciences, Tohoku University, Sendai 980-8578, Japan; 6 Department of Immunobiology, Institute of Development, Aging, and Cancer, Tohoku University, Sendai 980-8575, Japan; 7 Division of Pathogenic Biochemistry, Department of Bioscience, Institute of Natural Medicine, University of Toyama, Sugitani 2630, Toyama 930-0194, Japan; 8 Laboratory of Morphology and Image Analysis, Biomedical Research Center, Juntendo University Graduate School of Medicine, Tokyo 113-8421, Japan; 9 Department of Immuno-Gene Therapy, Mie University Graduate School of Medicine, 2-174 Edobashi, Tsu, Mie 514-8507, Japan; 10 Department of Oncology, Nagasaki University Graduate School of Biomedical Science, 1-12-4 Sakamoto, Nagasaki 852-8523, Japan; 11 Department of Hematology and Oncology, Icahn School of Medicine at Mount Sinai, 1470 Madison Avenue, New York, New York 10029, USA; 12 Atopy (Allergy) Research Center, Graduate School of Medicine, Juntendo University, Bunkyo-ku, Tokyo 113-8421, Japan; 13 Cancer Division, Garvan Institute of Medical Research, Darlinghurst, New South Wales 2010, Australia; 14 Immunology in Cancer and Infection Laboratory, QIMR Berghofer Medical Research Institute, Herston, 4006 Queensland, Australia; 15 School of Medicine, University of Queensland, Herston, 4006 Queensland, Australia

## Abstract

Genetic evolution that occurs during cancer progression enables tumour heterogeneity, thereby fostering tumour adaptation, therapeutic resistance and metastatic potential. Immune responses are known to select (immunoedit) tumour cells displaying immunoevasive properties. Here we address the role of IFN-γ in mediating the immunoediting process. We observe that, in several mouse tumour models such as HA-expressing 4T1 mammary carcinoma cells, OVA-expressing EG7 lymphoma cells and CMS5 MCA-induced fibrosarcoma cells naturally expressing mutated extracellular signal-regulated kinase (ERK) antigen, the action of antigen-specific cytotoxic T cell (CTL) *in vivo* results in the emergence of resistant cancer cell clones only in the presence of IFN-γ within the tumour microenvironment. Moreover, we show that exposure of tumours to IFN-γ-producing antigen-specific CTLs *in vivo* results in copy-number alterations (CNAs) associated with DNA damage response and modulation of DNA editing/repair gene expression. These results suggest that enhanced genetic instability might be one of the mechanisms by which CTLs and IFN-γ immunoedits tumours, altering their immune resistance as a result of genetic evolution.

## Abstract (web summary)

T cell mediated anti-tumour immune responses result in the emergence of an immune-resistant population in a process called immunoediting. Here, the authors show that immunoediting is associated with an increase in genomic rearrangements of tumour cells that requires both cytotoxic T cells and IFNγ exposure.

Immune responses are known to select (immunoedit) tumour cells displaying immunoevasive properties^1^,^2^,^3^,^4^. Both cytotoxic activity and IFN-γ production by CTL recognizing tumours are critical for cancer immunoediting, however, whether IFN-γ is anti-tumorigenic^5^,^6^,^7^,^8^,^9^,^10^,^11^ or pro-tumorigenic^12^,^13^,^14^,^15^,^16^ remains controversial^17^,^18^,^19^. DNA copy-number alterations (CNAs) are critical pathogenic events that drive tumour development^20^ and are involved in genetic evolution that confers malignant behaviour on cancer cells. Indeed, highly rearranged genomes harbouring many recurrent CNAs have been observed in human and mouse cancers^21^,^22^. A significantly higher level of CNAs was observed particularly in driver genes of genetically induced mouse non-small-cell lung carcinoma^23^,^24^. It was recently reported that majority of CNAs are acquired in short punctuated bursts at the earliest stages of tumour evolution^25^,^26^,^27^, possibly under the selective immunoediting pressure of CTL recognizing tumour-specific rejection antigens and acting on tumour cells^3^. Tumours derived from immunodeficient mice often express endogenous tumour-specific rejection antigens and these, or exogenous antigens genetically engineered into tumour cells, can be immunoedited during tumour development^3^,^4^. To address the mechanism by which IFN-γ contributes to cancer immunoediting and whether CTL/IFN-γ-mediated immunoediting influences CNAs, here, we examined tumours expressing immunogenic antigens in the context of cytotoxic T-cell (CTL)-mediated immunoediting *in vivo*. Our results indicate that exposure of tumours to antigen-specific IFN-γ-producing CTL results in tumour CNAs that correlate with immune resistance.

## Results

### HA-specific CTLs result in HA antigen loss

**Figure 1:**
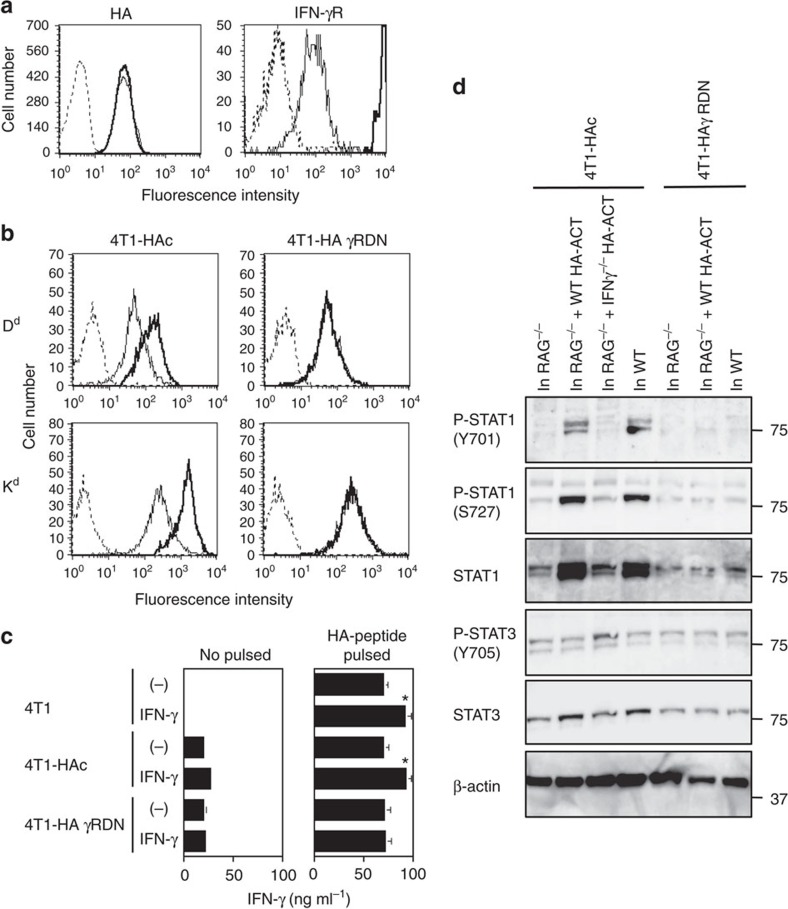
4T1-HAc cells respond to IFN-γ. (**a**) HA (left panel) and IFN-γRα chain (right panel) expression on 4T1-HAc (thin line) and 4T1-HAγRDN (thick line) cells were analysed by flow cytometry. Staining of 4T1-HAc and 4T-HAγRDN cells with isotype control mAb was indistinguishable (the level indicated by the dotted line). HA expression level on parental 4T1-HA cells was comparable to that on 4T1-HAγRDN and 4T1-HAc cells. (**b**) MHC class I expression on 4T1-HAc and 4T-HAγRDN cells was analysed by flow cytometry after 24 h culture with (thick lines), or without (thin line), IFN-γ. Staining of both cell populations with isotype control mAb was indistinguishable after the culture with or without IFN-γ (the level indicated by the dotted line). MHC class I expression level of parental 4T1-HA cells was comparable to that of 4T1-HAγRDN and 4T1-HAc cells and was similarly augmented by IFN-γ as for 4T1-HAc cells. (**c**) After incubation with or without HA peptide in the presence or absence of IFN-γ for 24 h, 4T1, 4T1-HAc, and 4T1-HAγRDN cells were co-cultured with HA-specific WT CTL for 24 h, then IFN-γ levels in the cell-free culture supernatants were determined by ELISA. Data are shown as mean±s.d. of three independently cultured cells. *P<0.05 as compared with the supernatant harvested from the culture of the same cells that were pre-incubated without IFN-γ by unpaired, two-tailed Student's *t*-test. (**d**) 4T1-HAc and 4T1-HAγRDN cells were inoculated into the same *RAG*^−/−^ and WT mice, and 10 days later *RAG*^−/−^ mouse was treated with HA-specific WT CTL. Five days after ACT, 4T1-HAc and 4T1-HAγRDN cells were isolated from the growing tumour mass. 4T1-HAc cells grown in *RAG*^−/−^ mouse treated with HA-specific IFN-γ^−/−^ ACT-treated (at day 10) were also collected at day 15. Phosphorylation of STAT1 and STAT3 in tumour cells was analysed by western blotting. Similar results were obtained in four experiments (**a**,**b**) and three experiments (**c**,**d**).

We first established single-cell-derived clones of 4T1 mammary tumours that express either influenza haemagglutinin (HA) antigen (4T1-HA), HA and a dominant-negative form (DN) of the IFN-γ receptor (IFN-γR) (4T1-HAγRDN) or HA and control vector (4T1-HAc). These 4T1-derived tumour clones expressed equivalent levels of HA and MHC class I, and equally induced HA-specific CTL *in vivo*, and equally re-stimulated HA-specific CTLs *in vitro*, despite the impaired IFN-γ responsiveness of 4T1-HAγRDN cells (Fig. 1a–c; Supplementary Fig. 1a–d). These results indicate the equivalent HA antigenicity of each 4T1 clone. The phosphorylation of STAT1 in 4T1-HAc cells, but not 4T1-HAγRDN cells, in response to adoptive wild type (WT), but not IFN-γ^−/−^, CTL transfer (ACT) into *RAG*^−/−^ mice demonstrated that 4T1-HA cells responded to IFN-γ produced by HA-specific CTL *in vivo* (Fig. 1d; Supplementary Fig. 1e,f).

**Figure 2:**
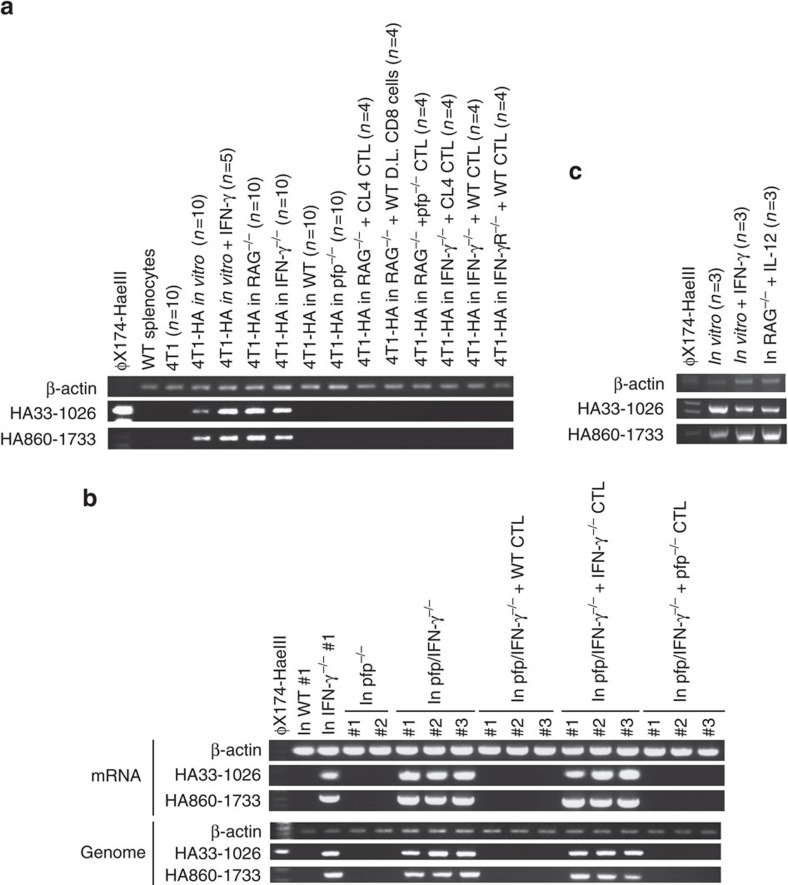
Integrated HA gene loss in 4T1-HA cells responding to IFN-γ and CTL i*n vivo.* (**a**) mRNA was prepared from WT splenocytes, 4T1 cells cultured *in vitro*, and representative HA-positive and HA-negative 4T1-HA cells isolated after the growth under the indicated conditions, then the indicated segments of HA gene were amplified by RT–PCR. RT–PCR was performed on every 4T1-HA cells independently, and the indicated number of PCR products were mixed and loaded in the respective groups. (**b**) Genome DNA and mRNA were prepared from 4T1-HA cells grown in the indicated mice for 25–35 days, and the indicated segments of the HA gene were amplified by RT–PCR and genomic PCR. (**c**) Genome DNA was prepared from three 4T1-HA cells grown independently i*n vitro* with or without IFN-γ or in IL-12-treated *RAG*^−/−^ mice for 30 days, and the indicated segments of the HA gene were independently amplified by genomic PCR. PCR products were mixed and loaded in the respective groups. Similar results were obtained in two independent experiments in all presented experiments (**a**–**c**).

A loss of tumour antigen expression has been reported in recurrent tumour cells resisting ACT^28^,^29^. To explore the impact of HA-specific CTL on the immunogenicity of 4T1-HA tumour cells, we analysed the HA expression of 4T1-HA cells after the *in vivo* growth under different immune selection conditions. While HA mRNA expression was stable in 4T1-HA cells after *in vitro* culture, or following growth in *RAG*^−/−^ or *IFN-γ*^−/−^ mice, HA mRNA was lost in all tested 4T1-HA cells grown *in vivo* in the context of IFN-γ producing HA-specific CTL over 25 days (Fig. 2a). Consistently, 4T1-HA and 4T1-HAc cells lost their surface HA protein expression upon *in vivo* exposure to IFN-γ-producing HA-specific CTLs (Supplementary Fig. 2a–c), and these 4T1-HA cells completely failed to stimulate HA-specific CTLs *in vitro* (Supplementary Fig. 2d). By contrast, 4T1-HAγRDN cells maintained HA protein expression and their antigenicity even following the growth in WT mice (Supplementary Figs 2b and 3a,b) and were more sensitive to ACT with HA-specific CTL compared with 4T1-HAc cells (Supplementary Fig. 3c). Of note, the introduction of STAT1 DN in 4T1-HA cells (4T1-HAS1DN cells) reduced the loss of HA antigenicity following CTL exposure (Supplementary Figs 1e and 4a–e), suggesting that 4T1-HA cells lose HA expression through an IFN-γR/STAT1-signalling pathway in response to IFN-γ produced by HA-specific CTL *in vivo*.

### IFN-γ-production is necessary for CTL-mediated HA gene loss

To further investigate the mechanisms underpinning loss of HA expression, we examined the status of the HA gene integrated into the tumour cell genome. While the HA gene remained intact in 4T1-HA cells grown in *IFN-γ*^−/−^ mice or *pfp/IFN-γ*^−/−^ mice, 4T1-HA cells grown in WT mice or *pfp*^−/−^ mice completely lost HA at both the level of mRNA and the genome (Fig. 2b). Importantly, ACT with WT or *pfp*^−/−^ CTL, but not *IFN-γ*^−/−^ CTL, into *pfp/IFN-γ*^−/−^ mice induced the loss of HA gene at the genome (Fig. 2b). By contrast, the HA gene was never lost in 4T1-HA cells cultured *in vitro* with recombinant IFN-γ or grown in *RAG*^−/−^ mice treated with repeated IL-12 administration to induce systemic IFN-γ production (Fig. 2c). Further, the HA gene was never lost in 4T1-HA cells co-cultured with *pfp*^−/−^ HA-specific CTL or WT CTL with perforin inhibitor, concanamycin A (CMA; Supplementary Fig. 3d), or in 4T1-HAγRDN or 4T1-HAS1DN cells grown in ACT-treated *RAG*^−/−^, *IFN-γ*^−/−^ or *IFN-γR*^−/−^ mice (Supplementary Fig. 4f).

These results suggest IFN-γ-producing HA-specific CTL within the tumour microenvironment are required for genomic rearrangements leading to the loss of the HA transgene in 4T1-HA cells. This loss of HA antigen may be one mechanism of many that contributes to immune evasion. To test if such HA gene loss could be a result of *in vivo* outgrowth of a very minor population within 4T1-HA cells lacking HA, we isolated and inoculated the cancer stem cell-like side population (SP) or main population (MP) of 4T1-HA cells into *RAG*^−/−^ or WT mice (Supplementary Fig. 5a,b; Supplementary Table 1). Even when the tumour developed from 50 cells of the SP of 4T1-HA cells, HA expression and gene were lost in WT mice, but not in *RAG*^−/−^ mice, similar to the tumours developed from the MP of 4T1-HA cells inoculated in WT mice (Supplementary Fig. 5c,d). These results suggested the loss of the HA transgene in immune-resistant 4T1-HA cells was critically dependent upon IFN-γ, and CTL-mediated cytotoxicity alone was not sufficient since ACT with IFN-γ-deficient HA-specific CTL, that have perforin-mediated cytotoxic activity intact, did not lead to HA gene loss. Moreover results suggest that loss of the HA transgene occurred during *in vivo* growth rather than as the result of the selective expansion of pre-existing HA gene negative cells within the 4T1-HA cells.

### IFN-γ-producing CTL results in CNAs in 4T1-HA tumour cells

**Figure 3:**
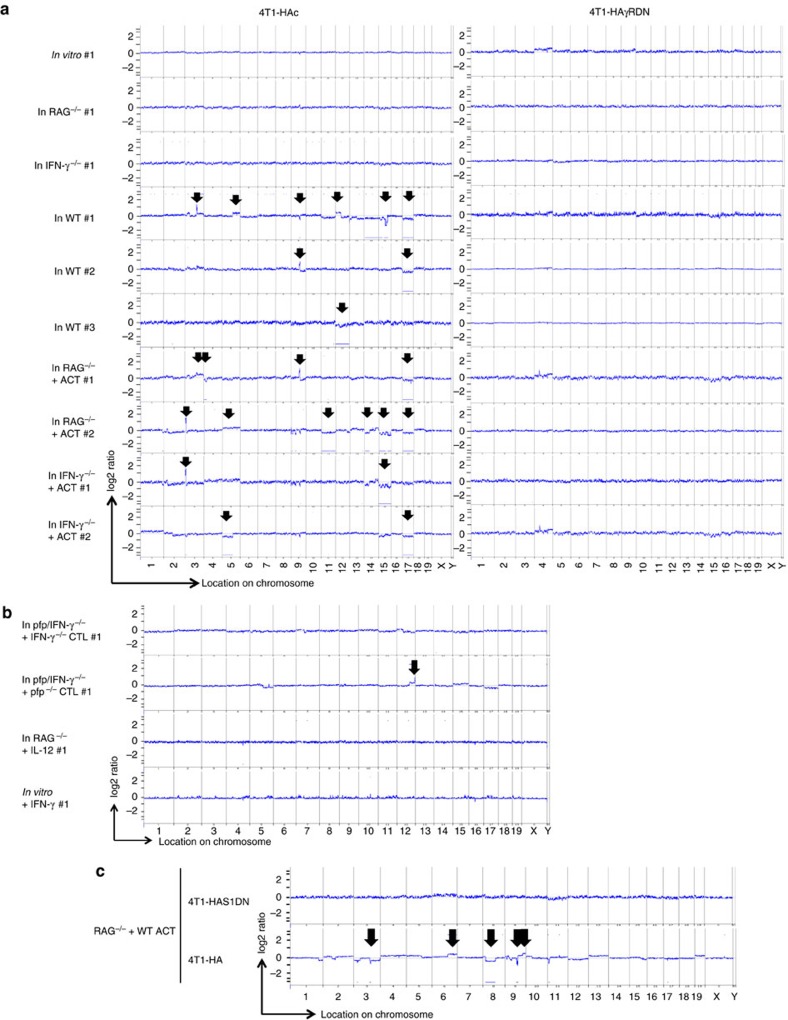
Genomic alteration in 4T1-HA cells responding to IFN-γ and CTL *in vivo.* Genomic DNA were prepared from 4T1-HAc and 4T1-HAγRDN cells that were isolated from the growing tumour mass in the indicated mice (>99% purity) (**a**) or 4T1-HAc cells isolated from the growing tumour mass in *pfp/IFN-γ*^−/−^ mice 25 days after the ACT with HA-specific *IFN-γ*^−/−^ or *pfp*^−/−^ CTL or in IL-12-treated *RAG*^−/−^ mice at 30 days or from 4T1-HA cells cultured with IFN-γ for 30 days in Fig. 2b,c (>99% purity) (**b**). Then, CNAs were examined by a-CGH used for s.c. inoculation as the reference sample. (**c**) 4T1-HA cells and 4T1-HAS1DN cells were inoculated into the same *RAG*^−/−^ mice that were treated with ACT on day 0. Genomic DNAs were obtained from both tumour cells prepared from the growing tumour masses 30 days after the tumour inoculation. Then, CNAs were examined by a-CGH. In a-CGH analysis, the tumour cells used for the s.c. inoculation are the reference sample. The positions showing significant CNA are indicated by the lines and arrows.

To further explore the possible contribution of genetic alteration to HA gene loss in 4T1-HA tumour cells, we performed array-based comparative genome hybridization (a-CGH) analysis of 4T1-HAc and 4T1-HAγRDN cells grown *in vitro* and *in vivo* (Fig. 3a; Supplementary Fig. 6a). Importantly, no significant genomic alterations were observed in either 4T1-HAc or 4T1-HAγRDN cells after one month of *in vitro* culture, indicating that the genomes of these cells were stable *in vitro*. By contrast, when these tumour cells were grown subcutaneously for one month under immunological pressure in immunocompetent WT mice, CNAs were observed in 4T1-HAc cells, but not 4T1-HAγRDN cells. Notably, although few CNAs were observed in 4T1-HAc cells grown in immune-deficient *RAG*^−/−^ mice or *IFN-γ*^−/−^ mice, ACT of HA-specific CTL into these mice resulted in increased CNAs. The patterns of genomic rearrangement were variable between resistant populations, consistent with increased genomic instability. Fluorescence *in situ* hybridization (FISH) analysis confirmed the peak of augmented expression in chromosome 3A1 of 4T1-HAc cells grown in ACT-treated *IFN-γ*^−/−^ mouse #1 and ACT-treated *RAG*^−/−^ mouse #2 (Supplementary Fig. 6b,c). CNAs appear not to be simply a result of aneuploidy, since the chromosome number of these two 4T1-HA cell populations losing the HA transgene was not significantly different compared with 4T1-HAc cells grown *in vitro*, *in RAG*^−/−^ *or IFN-γ*^−/−^ mice (Supplementary Fig. 6d). FISH analysis demonstrated that a single HA transgene was integrated into chromosome 17B of the parental 4T1-HA cells (Supplementary Fig. 6e), and copy-number losses were observed within chromosome 17 in five out of the seven 4T1-HA cell populations losing the HA gene (Fig. 3a). In accordance with the HA gene loss, ACT with HA-specific *pfp*^−/−^ CTL, but not *IFN-γ*^−/−^ CTL, results in CNAs in 4T1-HA cells grown in *pfp/IFN-γ*^−/−^ mice (Fig. 3b; Supplementary Fig. 6f), and CNAs were not observed in 4T1-HA cells either grown in *RAG*^−/−^ mice repeatedly treated with IL-12, cultured *in vitro* with IFN-γ (Fig. 3b), or 4T1-HAS1DN cells grown in ACT-treated *RAG*^−/−^ mice (Fig. 3c).

Moreover, kinetic analysis indicated that CNAs accumulate during immunological selection (Supplementary Fig. 7a) and lead to the intra-tumour heterogeneity accompanied by tumour antigen gene loss in 4T1-HA cells (Supplementary Fig. 7b). These results suggest that HA gene loss occurs when CTL and CTL-derived IFN-γ resulted in increased CNAs in 4T1-HA cells in the tumour microenvironment.

### CNAs in OVA-expressing EG7.1 cells exposed to CTL *in vivo*

**Figure 4:**
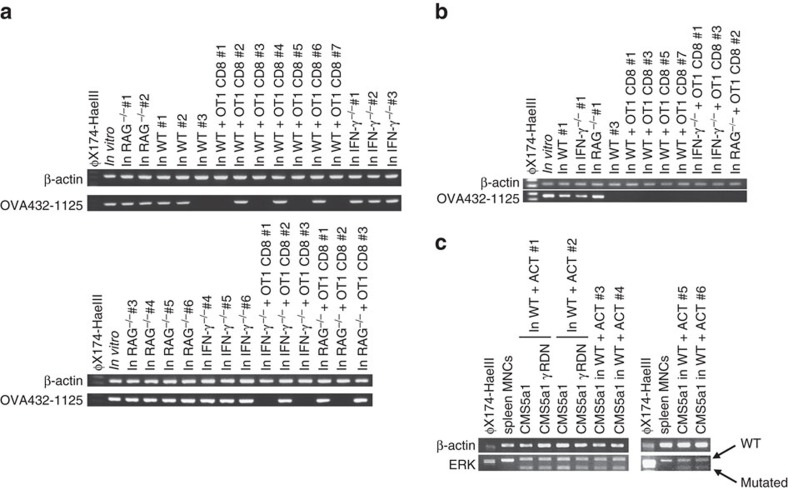
Antigen gene expression in EG7.1 cells and CMS5a1 cells after the exposure to tumour-specific CTL *in vivo*. (**a**,**b**) EG7.1 cells were inoculated into the indicated mice, and some mice were treated with ACT of OVA-specific OT-1 CTLs on day 0. mRNA and genome DNA were prepared from EG7.1 cells grown 25–35 days. The indicated segment of the OVA gene, that contains H-2K^b^-restricted CTL epitope targeted by OT-1, was amplified by RT–PCR (**a**) or genomic PCR (**b**). (**c**) mRNA was prepared from CMS5a1 cells isolated from the growing tumour mass in the indicated mice. ERK gene was amplified by RT–PCR, then, PCR products were digested by *Sfcl* restriction enzyme that selectively cleaves mutated ERK, but not wild type ERK2. Similar results were obtained in two independent experiments in all presented experiments (**a**–**c**).

**Figure 5:**
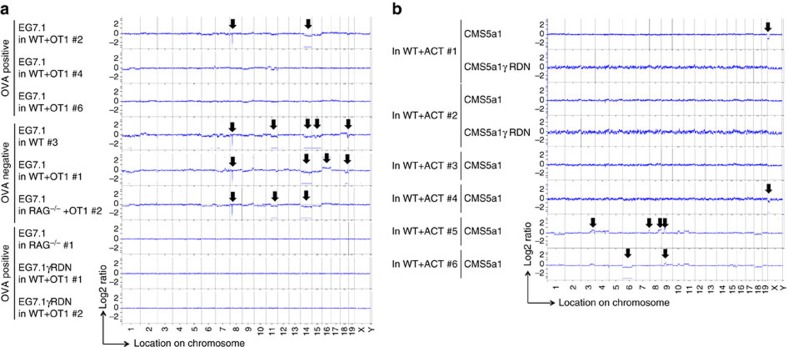
Genetic alteration in EG7.1 cells and CMS5a1 cells after the exposure to tumour-specific CTL *in vivo*. (**a**) Genomic DNAs were prepared from EG7.1 cells isolated from the growing tumour mass in the indicated mice (Fig. 4a,b) and EG7.1γRDN cells isolated from the growing tumour mass in OT-1-treated WT mice 25 days after tumour inoculation (Supplementary Fig. 8d). Then, CNAs were examined by a-CGH employing tumour cells used for s.c. inoculation as the reference sample. (**b**) Genomic DNAs were prepared from CMS5a1 cells isolated from the growing tumour mass in the indicated mice as in Fig. 4c and Supplementary Fig. 9a–c. Then, CNAs were examined by a-CGH employing tumour cells used for s.c. inoculation as the reference sample. The positions showing significant CNA are indicated by the lines and arrows.

To clarify whether CNA induction and/or tumour-antigen loss was a cell-type or antigen-specific effect, we next established a single cell-derived clone (EG7.1) from OVA-expressing EG7 lymphoma cells and an EG7.1-derived single cell clone expressing DN of IFN-γR (EG7.1γRDN; Supplementary Fig. 8a,b). The expression of IFN-γR DN augmented the susceptibility to ACT in *RAG*^−/−^ mice (Supplementary Fig. 8c), similar to the observation made in 4T1-HA cells. While somewhat less EG7.1 cells lost their OVA mRNA expression and OVA gene in genome through an IFN-γ-mediated immunoediting process (Fig. 4a,b), marked CNAs were frequently observed in EG7.1 cells exposed to CTL *in vivo* (Fig. 5a), but not in EG7.1 cells grown in *RAG*^−/−^ mice or OVA-expressing EG7.1γRDN cells grown in WT mice treated with OT-1 CTL (Fig. 5a and Supplementary Fig. 8d).

### CNAs in CMS5a1 tumour cells exposed to CTL *in vivo*

To study the effect of CTLs recognizing endogenous antigens, we employed a single cell-derived clone (CMS5a1) from CMS5 cells that express spontaneously mutated extracellular signal-regulated kinase (ERK) as the endogenous tumour antigen^29^ and a CMS5a1-derived single cell clone expressing DN of IFN-γR (CMS5a1γRDN). Again, ACT with mutated ERK (mERK)-specific CTLs induced CNAs in four of six tested CMS5a1 cells, but not in CMS5a1γRDN cells (Fig. 5b). Interestingly, the frequency of CNA induction was increased when ACT treatment was more extended (Fig. 5b; Supplementary Fig. 9a–c). The copy-number reduction demonstrated by a-CGH analysis was confirmed by the quantitative reverse transcription (RT)–PCR for *GM1374* that is located at chromosome XA1.1 (Supplementary Fig. 9d). Unexpectedly, all tested CMS5a1 cells maintained expression of mERK mRNA (Fig. 4c). Given that several immunoedited EG7.1 and CMS5a1 cells still expressed their antigens (Fig. 4a,c), it is likely that other mechanisms and/or modification of other gene expression by CNAs contributes to immune evasion in resistant subclones following exposure to IFN-γ producing antigen-specific CTLs. Loss of the CTL-targeted antigen might be the most efficient method to escape from tumour-specific CTLs. However, genomic instability associated with *in vivo* immunological pressure is consistently observed across multiple tumour cell types, vectors, and antigens, suggesting a fundamental mechanism driving adaptive evolution under immune selection. The distinct results with the CMS5a1 cells suggest that perhaps the transfected antigen systems show bias towards antigen loss or longer timeframes are required with endogenous neo-antigens to witness a higher frequency of antigen loss.

### IFN-γ-producing CTL effect on DNA damage and repair pathways

**Figure 6:**
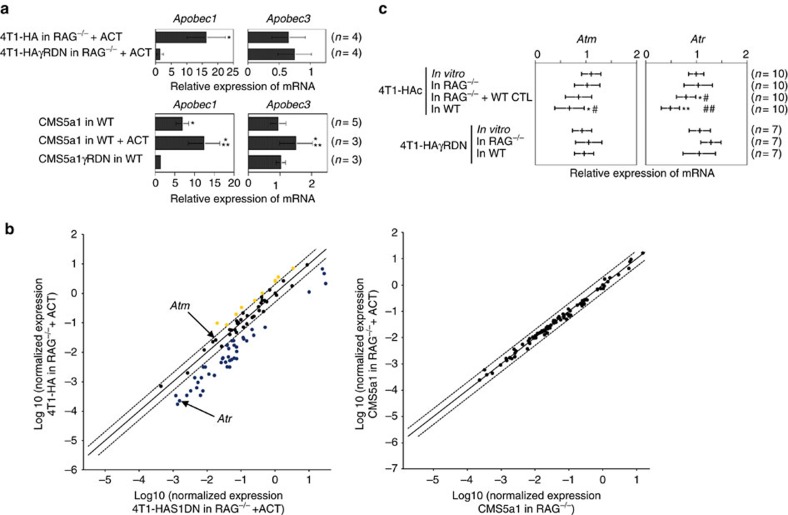
Altered expression of genes implicated in DNA repair and maintenance in tumour cells exposed to CTL-mediated immunoediting. (**a**) Expression of *Apobec 1* and *3* genes in 4T1-HA and 4T1-HAγRDN cells growing in ACT-treated *RAG*^−/−^ mice or CMS5a1 and CMS5a1γRDN cells growing in WT or ACT-treated WT mice. The gene expression was normalized to β*-actin* levels, and the relative expression compared with the respective cells grown in *RAG*^−/−^ mice (in upper panels) or respective *in vitro* cultured cells (in lower panels). Results are indicated as the average±s.d. of the results obtained from the experiments using the numbers of tumour cells indicated in parentheses. *P<0.05 compared with IFN-γR DN expressing respective cells; **P<0.005 compared with CMS5a1 cells grown in WT mice. Both are analysed by unpaired, two-tailed Student's *t*-test. Similar results were obtained in two independent experiments. (**b**) mRNA was prepared from freshly isolated 4T1-HA and 4T1-HAS1DN cells grown in the same ACT-treated *RAG*^−/−^ mice (*n*=3 each) or CMS5a1 cells grown in *RAG*^−/−^ or ACT-treated *RAG*^−/−^ mice (*n*=3 each). Expression of DNA repair genes was examined by quantitative RT–PCR array. (**c**) mRNA was prepared from freshly isolated 4T1-HAc and 4T1-HAγRDN cells growing *in vitro*, in *RAG*^−/−^, WT HA-specific CTL-treated *RAG*^−/−^, and WT mice. Double strand DNA repairing protein kinase ataxia-telangiectasia and Rad3 related, *Atr*, and protein kinase ataxia-telangiectasia mutated, *Atm*, gene expression was examined by quantitative RT–PCR. The gene expression was normalized to *Gapdh* levels, and the relative expression compared with the mean value of the *in vitro* growing tumour samples is presented. Results are indicated as the average±s.d. of the results obtained from the experiments using the numbers of tumour cells indicated in parentheses. *P<0.05 compared with cells *in vitro*; **P<0.005 compared with cells *in vitro*; ^#^*P*<0.05 compared with cells in *RAG*^−/−^; ^##^*P*<0.005 compared with cells in *RAG*^−/−^. All are analysed by unpaired, two-tailed Student's *t*-test.

**Figure 7:**
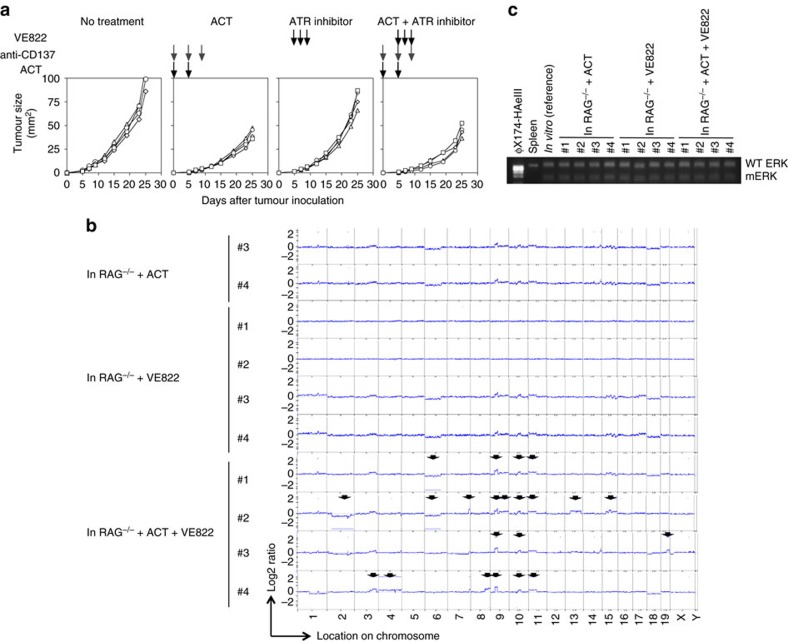
CNAs induced in CMS5a1 cells in *RAG*^−/−^ mice treated with ATR inhibitor and WT ACT. (**a**,**b**) CMS5a1 cells were inoculated into *RAG*^−/−^ mice, and some mice were treated with CD8 T cells prepared from DL of CMS5a1-bearing WT mice that were treated with anti-CD137 mAb as indicated by the black arrows on day 0 and 5. These ACT-treated mice were also treated with anti-CD137 mAb to activate CTL on day 0, 5 and 9 as indicated by the grey arrows. Some mice were treated with ATR inhibitor, VE822, on day 5, 7 and 9 as indicated by the black arrows. Tumour growth was measured and tumour cells were isolated 25 days after tumour inoculation (**a**). Genomic DNA and mRNA were prepared from CMS5a1 cells isolated from the tumour mass on day 25. Then, CNAs were examined by a-CGH employing tumour cells used for s.c. inoculation as the reference sample (**b**). The positions showing significant CNA are indicated by the lines and arrows. mRNA of ERK gene was amplified by RT–PCR, then, PCR products were digested by *Sfcl* restriction enzyme that selectively cleaves mutated ERK, but not wild type ERK2 (**c**). Concerning tumour growth and HA expression at RNA level, similar results were obtained in two independent experiments.

We observed a variable pattern of expression of genes implicated in DNA repair and maintenance by quantitative RT–PCR. The Apobec family is implicated in tumour diversity and subclonal evolution^30^. *Apobec3* expression was reported to be positively regulated by STAT1 (ref. 31). Consistently, *Apobec3,* as well as *Apobec1,* expression was increased in 4T1-HA and CMS5a1 cells compared with controls expressing IFN-γR DN, and gene expression in CMS5a1 cells was augmented upon ACT (Fig. 6a). Several other genes implicated in DNA repair and maintenance were reduced in 4T1-HA cells compared with 4T1-HAS1DN cells grown in ACT-treated *RAG*^−/−^ mice (Fig. 6b). However, DNA repair gene expression in CMS5a1 cells grown in *RAG*^−/−^ mice was not significantly modulated by ACT (Fig. 6b), but double strand breaks (measured by the number of phospho-Histone H2A.X (Ser139)(γH2A.X)-foci) were significantly increased in CMS5a1 cells, but not in CMS5a1γRDN cells, grown in *RAG*^−/−^ mice upon ACT (*P*<0.05 by unpaired, two-tailed Student's *t*-test)(Supplementary Fig. 9e,f), consistent with increased genomic instability. A reproducible reduction in expression of the double strand DNA damage-sensing gene, *Atr*, was confirmed by quantitative RT–PCR in 4T1-HA cells (Fig. 6c). Inactivation of ATM/ATR was reported to promote chromosomal instability^32^. Thus, we examined the effect of a small molecule inhibitor of ATR (VE822) on immune-induced genomic instability (Fig. 7a). Significant CNAs were induced in CMS5a1 cells upon ACT combined with VE822 treatment (Fig. 7b), which were not observed in CMS5a1 cells grown in ACT-treated or VE822-treated *RAG*^−/−^ mice. All tested CMS5a1 cells retained their endogenous tumour antigen (mERK) expression (Fig. 7c). Collectively, these results suggest that CTL and CTL-derived IFN-γ may induce genomic instability through the modulation of DNA damage responses and repair pathway in tumour cells *in vivo*.

### CNAs in IFN-γ-producing 4T1-HA cells

**Figure 8:**
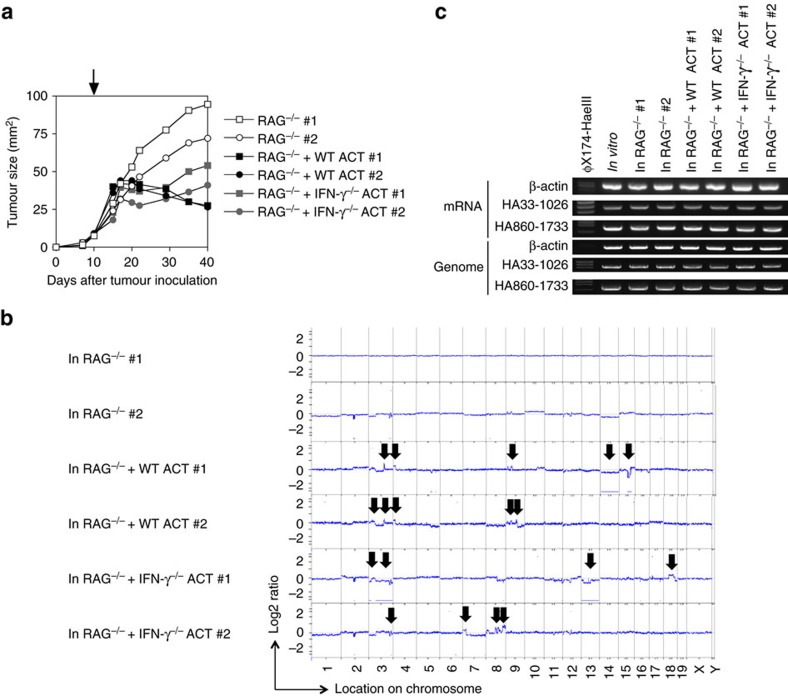
CNAs induced in IFN-γ-producing 4T1 tumours in *RAG*^−/−^ mice treated with WT or *IFN-γ*^−/−^ ACT. 5 × 10^5^ of 4T1-HA cells producing high amount of IFN-γ (4T1-HAIFNγTf) were inoculated into *RAG*^−/−^ mice. As indicated by arrow, when palpable tumours developed after 10 days, mice were received T cells (5 × 10^7^ per mice) obtained from draining lymph node of WT or *IFN-γ*^−/−^ mice that were inoculated with 4T1-HAIFNγTf cells 7 days before the sacrifice. Tumour cells were isolated from tumour mass 30 days after ACT (**a**), and genomic DNAs and mRNAs were prepared. CNAs were examined by a-CGH employing tumour cells used for s.c. inoculation as the reference sample (**b**). The positions showing significant CNA are indicated by the lines and arrows. The indicated portion of the HA gene were amplified by RT–PCR and genomic PCR (**c**). Concerning tumour growth and HA expression at RNA level, similar results were obtained in two independent experiments.

To further address the importance of microenvironment on CTL-induced genetic instability, we inoculated IFN-γ-overexpressing 4T1-HA cells into *RAG*^−/−^ mice treated with CTL. We established IFN-γ-producing single cell-derived clones (4T1-HAIFNγTf) from 4T1-HA cells. 4T1-HAIFNγTf produced >1.1 μg ml^−1^ of IFN-γ when cells were cultured *in vitro* for 16 h at 5 × 10^5^ cells per 200 μl. 4T1-HAIFNγTf cells grew slowly *in vitro* and in *RAG*^−/−^ mice compared with 4T1-HA cells, and never progressively grew when 2 × 10^6^ 4T1-HAIFNγTf cells were inoculated in WT mice (*n*=6). We obtained genomic DNA and RNA from 4T1-HAIFNγTf cells isolated from tumour masses in *RAG*^−/−^ mice 30 days after the treatment with draining lymph node T cells (DL) that were harvested from 4T1-HAIFNγTf cells-inoculated into WT or *IFN-γ*^−/−^ mice (Fig. 8a). As expected, CNAs or HA gene loss were not observed in 4T1-HAIFNγTf cells isolated from the tumour masses in *RAG*^−/−^ mice (Fig. 8b,c). Further, marked CNAs were observed in 4T1-HAIFNγTf cells that were obtained from tumour masses in *RAG*^−/−^ mice treated with CD8^+^ T cells of WT or *IFN-γ*^−/−^ DL cells (Fig. 8b), although these cells retained the HA RNA and HA gene (Fig. 8c). These results suggest that CNAs can be induced by antigen-specific CTLs that are impaired in IFN-γ production, if ectopic IFN-γ is released by tumour cells. Thus, to induce CNAs in tumour cells, IFN-γ is critical, but does not have to be produced necessarily by tumour-specific CTL. Our findings also show that CNAs induction is not simply replicated by supplementing IFN-γ within tumour microenvironment, rather the genetic instability is augmented in tumour cells only when IFN-γ and CTLs co-exist in tumour microenvironment.

## Discussion

IFN-γ is regarded to play critical roles in anti-cancer immune responses by augmentation of MHC Class I expression, growth arrest^7^, post-proteasomal trimming of antigen epitopes^8^ and recruitment of effector cells^9^. Moreover, the transcription factor interferon regulatory factor (IRF)-1 was reported to manifest tumour-suppressor activity in tumour cells^10^. Consistently, we have also reported important roles of IFN-γ in tumour-rejecting CTL functions^33^ and NK cell-mediated anti-metastatic effects^34^,^35^. By contrast, it was also reported that IFN-γ appreciably contributes to aberrant DNA methylation^16^, tumour initiation^12^, survival and outgrowth^13^. Another very recent study showed that prolonged IFN signalling in tumour cells increased resistance to immune checkpoint blockade through multiple inhibitory pathways^36^. Notably, it was reported that IFN-γ promotes an immune suppressive microenvironment during MCA-induced carcinogenesis, but conversely promotes anti-tumour immune responses against transplanted MCA-induced sarcomas^19^. In MCA-induced fibrosarcoma models, immunoediting has been confirmed^3^ and IFN-γ responsiveness of tumour cells was reported to be critical to anti-tumour immune responses^11^. Thus, the roles of IFN-γ in tumour development and growth are variable and complex depending upon the tumour model, phase of tumour development, and success of immune selection pressure. Moreover, tissue microenvironment (niche) appears to be important for the biological and genetic progression of malignancy^37^. Our data herein suggest that tumour cells adapt in the context of host immune responses and the microenvironment. Tumours develop heterogeneity and progress to escape variants with greater malignancy^38^, not only by epigenomic or post-transcriptional alterations, but also by promoting genetic instability with CNAs.

The genomic instability induced by CTL and IFN-γ during tumour progression in this study is in the context of tumour adaptation rather than initiation. These mutations in some circumstances confer an immunoevasive growth advantage, metastatic potential and therapeutic resistance^24^. Interestingly, genomic instability was consistently observed in all-tested tumour cells, however, loss of target antigen was not observed in CMS5a1 cells. This suggests that increased genetic diversity generated by immunological genomic instability favours the stochastic emergence of resistant genotypes, which is sometimes associated with loss of antigen, but is alternatively sometimes due to other unknown mechanisms. The preservation of the ERK mutation in CMS5a1 cells may be due to counter-selection for the growth advantage associated with this growth signalling kinase. Presumably there are multiple possible routes to immune evasion, the favouring of which is dependent on a balance of selective pressures and stochastic events.

We show that CTL/IFN-γ promoted genetic alteration in tumour cells and the frequency of such genetic alteration was associated with their immunogenicity. What is the molecular link between CD8^+^ T cells and IFN-γ production to genomic alteration in tumour cells? Understanding this final molecular step is critical, and identifying such would represent a critical advance in the field. We hypothesize that these processes are relevant to the immunoediting process during an immune:cancer equilibrium^1^, when many de novo tumours express non-self endogenous rejection antigens^3^,^4^. CTL-induced alteration of genetic diversity might arise relatively early during carcinogenesis, generating a ‘Cambrian' explosion of subclones characterized by gross genomic instability. Consistent with this, a single-cell DNA sequencing method recently suggested that large-scale structural changes in the genome, rather than point mutations, possibly occur early in tumour development^39^. It was also recently reported that majority of CNAs were acquired in short punctuated bursts at the earliest stages of tumour evolution^25^,^26^,^27^. Other mechanisms may apply at later phases of tumour progression, where CTL-secreted IFN-γ induces stem cell proliferation^15^ and PD-L1 expression on tumour cells^14^. It was also reported that melanoma cells reversibly downregulate melanocytic lineage antigens responding to TNF-α produced by CTL following therapy^40^. Together, these data suggest a dynamic multifactorial interaction between cancer cells and anti-tumour CTL throughout tumour development, despite the fact that anti-tumour CTL are generally critical suppressors of tumour development.

Tumours often eventually relapse after transient suppression following ACT therapy with tumour-associated antigen-specific CTL^41^, suggesting that tumour cells are able to acquire resistance by downregulating their immunogenicity^28^,^40^,^42^ or by inducing T-cell tolerance^14^,^43^. The results presented here suggest that the induction of genomic instability may lead to resistance to immunotherapies. On the other hand, neo-antigen expression, mutations in driver genes, and CNAs of gene loci containing immune regulators were associated with the expression of immune cytolytic molecules in human tumours^44^. Considering tumours that are susceptible to immune checkpoint-targeting therapies bear higher levels of somatic mutations possibly due to the exposure to strong carcinogens^45^, genomic alterations not only result in the induction of neo-antigens that can drive immune responses, but paradoxically drive immune-evasion.

CTL expressing high-avidity antigen-specific T-cell receptor recognizing antigen with high affinity for MHC Class I are critical for the effective immune therapies^46^,^47^. Such strong immunotherapies also induce antigen-negative variants, thus, combination with additional therapies is required to overcome the escape^47^. Our findings possibly support a theoretical advantage of combining immune therapies targeting ‘oncoantigens' that play critical roles for tumour cell maintenance and growth^48^, with therapies targeting genomic repair and maintenance mechanisms. The increased dependency of cancer cells on genomic instability following exposure to immunotherapies may render cancer cells more susceptible to DNA damage-inducing chemotherapies and/or radiotherapies, or the increasing suite of drugs targeting DNA repair and maintenance.

## Methods

### Mice

Six- to 8-week-old wild-type (WT) BALB/c and C57BL/6 mice were from Charles River Japan Inc. (Yokohama, Japan) and The Walter and Eliza Hall Institute of Medical Research (Melbourne, Australia). BALB/c IFN-γ-deficient (*IFN-γ*^−/−^), TNF-related apoptosis-inducing ligand (TRAIL)-deficient (*TRAIL*^−/−^), perforin-deficient (*pfp*^−/−^), and perforin- and IFN-γ-deficient (*pfp/IFN-γ*^−/−^) mice were derived as described previously^33^,^34^,^49^. BALB/c Rag-2-deficient (*RAG*^−/−^) mice were provided from the central Institute for Experimental Animals (Kawasaki, Japan)^50^. BALB/c IFN-γ receptor deficient (*IFN-γR*^−/−^) mice were derived from Jackson Laboratory (Bar Harbor, ME, USA)^51^. BALB/c H-2K^d^-restricted HA-specific TCR transgenic (CL4) mice^52^ were bred at the Peter MacCallum Cancer Centre. C57BL/6 Rag-2-deficient (*RAG*^−/−^) and H-2K^b^-restricted OVA-specific TCR transgenic (OT-1) mice were bred at Juntendo University^50^. H-2K^d^-restricted mutated ERK2 kinase protein (mERK)-specific TCR transgenic (DUC18) mice were bred at Mie University^29^. All mice were maintained under specific pathogen-free conditions and used in accordance with the institutional guidelines of Juntendo University, the Peter MacCallum Cancer Centre or Mie University. Number of female mice used for the experiments was decided based on historical controls. No mice were excluded based on pre-established criteria in this study and no active randomization or blinded classification was applied to experimental groups. All experiments were approved by Juntendo University, the Peter MacCallum Cancer Centre or Mie University. The variance was similar between groups when applying statistical analysis in each experiment.

### Tumour cells

4T1 mammary tumour cells (purchased from ATCC) expressing the influenza HA gene were generated by the retroviral transfection with MSCV-IRES-GFP vector containing HA cDNA from the Mount Sinai strain of the PR8 influenza virus^52^. HA protein expression on the cell surface was confirmed by flow cytometric analysis using HA-specific mAb, H18 (ref. 52), and PE- or eFluor 660-conjugated F(ab')_2_ fragment of goat anti-mouse IgG polyclonal antibody (eBioscience, San Diego, CA). Single cell-derived clone cells were established by single cell cloning following cell sorting using FACS aria (BD Bioscience, San Jose, CA). 4T1-HA cells expressing high level of the dominant-negative form (DN) of mouse IFN-γ receptor (IFN-γR) α chain (IFN-γR1) were established by the transfection with the pEF2.muγR plasmid containing the truncated murine IFN-γRα chain cDNA^35^,^53^. Control 4T1-HA cells (4T1-HAc) were established by transfection with the empty pEF2 vector. IFN-γR1 expression levels were examined by flow cytometric analysis using biotin-conjugated rat anti-mouse CD119 mAb (2E2) and PE-conjugated streptavidin (eBioscience). Then, single cell-derived clone cells were established as described above. Expression plasmids (pCAGGS-Neo) containing DN of STAT1 cDNA was kindly provided by Dr Koichi Nakajima (Department of Immunology, Osaka City University Graduate School of Medicine, Osaka, Japan)^54^. These were transfected into 4T1-HA cells using Amaxa Nucleofector (Lonza, Basel, Switzerland) according to the manufacturer's instructions to obtain 4T1-HA cells expressing high levels of DN of STAT1 (4T1-HAS1DN). Control 4T1-HA cells (4T1-HAPCAV) were established by transfection with the pCAGGS-Neo vector. Single cell-derived clone cells were established by single cell cloning with geneticin (G418), and overexpression of DN STAT1 was confirmed by RT–PCR and immunoblot as previously described^54^. 4T1-HA cells expressing high level of IFN-γ were established by retroviral transfection using the pMXs-IRES-Puro vector harbouring the mouse IFN-γ gene. Single cell-derived clone cells (4T1-HAIFNγTf) were established and abundant IFN-γ production was confirmed using mouse IFN-γ specific enzyme-linked immunosorbent assay (ELISA) kit (Quantikine; R & D systems, Minneapolis, MN) according to the manufacturer's instructions. EG7.1 cells were established from EG7 cells (purchased from ATCC) by single cell cloning followed by the confirmation of stable OVA gene expression by RT–PCR. EG7.1 cells expressing high levels of DN mouse IFN-γR1 (EG7.1γRDN cells) were established as described above. Single cell-derived clone cells were established by single cell cloning as described above. CMS5a1 cells were established from CMS5a cells (that were kindly provided from Dr Hiroshi Shiku, Mie University, Mie, Japan) by single-cell cloning followed by the confirmation of stable mERK gene expression by RT–PCR. CMS5a1 cells expressing high levels of DN mouse IFN-γR1 (CMS5a1γRDN cells) were established as described above. Single-cell-derived clone cells were established by single-cell cloning as described above. All tumour cells used in this study were tested and authenticated negative for mycoplasma contamination.

### Flow cytometric analysis

Flow cytometric analyses were performed on FACSCalibur (BD Bioscience) following Immunofluorescence staining^34^. PE-conjugated anti-mouse H-2K^d^ mAb (SF1-1.1), anti-mouse H-2D^d^ mAb (34-2-12) (BD Bioscience), and isotype-matched control mAbs (eBM2a; eBioscience) were used to examine MHC class I expression levels on 4T1-HA and 4T1-HA IFNγRDN cells.

### Transplantation and preparation of tumour cells

Tumour cells (2–5 × 10^5^ per mice) were subcutaneously (s.c.) inoculated into the mice and the tumour size was measured periodically with a caliper as the product of two perpendicular diameters (mm^2^). Single-cell suspensions from solid tumours were prepared using collagenase (Wako Pure Chemicals, Osaka, Japan)^55^, then CD45^+^ cells and CD31^+^ cells were depleted using anti-PE MicroBeads and mouse CD45 MicroBeads on autoMACS (Miltenyi Biotec, Bergisch Glabach, Germany) following incubation with PE-conjugated anti-mouse CD31mAb (MEC13.3; BioLegend) according to the manufacturer's instructions. Freshly isolated tumour cells (purity>90%) were used for the analysis of genome maintenance factors by RT–PCR and flow cytometric analysis. Isolated tumour cells were cultured for >10 days *in vitro* and HA expression was confirmed by flow cytometric analysis. These were then used for the co-culture with lymphocytes, transduced HA or OVA gene expression analysis by RT–PCR, or genomic DNA analysis (purity>99%). In some experiments, 4T1-HA cells were cultured *in vitro* with 100 ng ml^−1^ of IFN-γ, 10% cell-free supernatant of concanavallin A (Con A) (Sigma, St Louis, MO) stimulated WT splenocytes (5 μg ml^−1^), 10% cell-free supernatant of CL4 CTL co-cultured with HA-pulsed WT mouse bone marrow-derived dendritic cells (BMDC; CD8^+^ cells: BMDC=10:1) for 5 days in the presence of HA peptide (1 μg ml^−1^) and IL-2 (200 ng ml^−1^), or *pfp*^−/−^ HA-specific CTL for 25 or 30 days (4T1-HA cells: CTL=10:1) or WT CTL with perforin inhibitor, concanamycin A (CMA; 50 nM; Wako Pure Chemicals), (4T1-HA cells: CTL=10:1) for 60 days. In some experiments, tumour cells were pulsed with H-2K^d^-restricted HA epitope peptide (^533^IYSTVASSL^541^; Invitrogen, Carlsbad, CA, USA; 1 μg ml^−1^) for 24 h before the co-culture and used as stimulator cells for HA-specific CTL.

### Induction of HA-specific or OVA-specific CTL

BMDC were prepared form BALB/c WT mice with granulocyte/macrophage-colony-stimulating factor (eBioscience)^56^, and cultured with LPS (Sigma, St. Louis, MO; 2 μg ml^−1^) and H-2K^d^-restricted HA epitope peptide (Invitrogen; 1 μg ml^−1^) overnight in RPMI-1640 (Nissui Pharmaceutical, Tokyo, Japan) supplemented with 0.2 mM L-glutamine (Wako), 25 mM NaHCO_3_ (Wako), 10% heat-inactivated fetal calf serum (FCS; JRH biosciences, Lenexa, KA), and 5 × 10^−5^ M β2-mercaptoethanol (Wako) at 37 °C in a 5% carbon dioxide humidified atmosphere^57^. The nylon non-adherent cells were enriched from freshly isolated splenic MNCs of CL4 mice using a nylon-wool column (Wako Pure Chemicals, Osaka, Japan), and cells (2.5 × 10^6^ per ml) were stimulated with HA-pulsed WT mice-derived BMDC (2.5 × 10^5^ per ml) in the presence of HA peptide (1 μg ml^−1^) and IL-2 (200 ng ml^−1^; eBioscience). When WT, *pfp*^−/−^ or *IFN-***γ**^−/−^ mice were used, 4T1, 4T1-HAc, 4T1-HA**γ**RDN or 4T1-HA cells (2 × 10^6^ cells) were i.p. inoculated into the mice, then nylon non-adherent cells were prepared from splenic MNCs 7 days later and co-cultured with HA-pulsed WT mice-derived BMDC as described above. IFN-**γ** (100 ng ml^−1^; eBioscience) was supplemented into the culture for the *in vitro* stimulation of *IFN-**γ***^−/−^ mouse-derived nylon non-adherent cells. After 7 days of co-culture, cells were harvested and CD8^+^ cells were purified by CD8α^+^ T-cell isolation kit on autoMACS (Miltenyi Biotec) according to the manufacturer's instructions. Flow cytometric analysis demonstrated the CD8^+^ cell population to be more than 95% pure. To induce OVA-specific CTL, we used B6 WT mice for BMDC preparation, H-2K^b^-restricted OVS epitope peptide (^257^SIINFEKL^264^; Invitrogen) and OT1-mice for splenic MNCs preparation.

### Preparation of tumour draining lymph node (DL) cells

Ten days after the inoculation of 4T1 or 4T1-HA cells (2 × 10^5^ per foot) into both footpads of BALB/c mice, CD8^+^ T cells were prepared from popliteal lymph node cells by CD8α^+^ T-cell isolation kit using autoMACS (Miltenyi Biotec) according to the manufacturer's instructions. Flow cytometric analysis demonstrated CD8^+^ cell population to be more than 95% pure.

### Adoptive cell transfer therapy (ACT)

Prepared cells were transferred intravenous (i.v.) into the mice (5 × 10^6^–2 × 10^7^ cells per mice) on the same day as tumour inoculation, 7 and/or 14 days after tumour inoculation, or 5, 10, 14 and 18 days after tumour inoculation. In the experiments using EG7.1 cells, prepared OT-1 cells were transferred i.v. into the mice (3 × 10^6^ cells per mice) on the same day as tumour inoculation. In some experiments, WT mice-derived splenocytes were stimulated with Con A (Sigma; 5 μg ml^−1^) for 5 days *in vitro*, then whole cells or isolated CD8^+^ cells were transferred into the mice on the same day as tumour inoculation. Some *RAG*^−/−^ mice were treated with IL-12 (50 ng per mice; kindly provided by Genetics Institute, Andover, MA) or PBS every 2 days after tumour inoculation.

In the experiments using CMS5a1 cells (presented in Figs 4c and 5b, Supplementary Fig. 9a,b; Fig. 6a,b), purified CD8^+^ T cells from spleen of DUC17 mice were transferred i.v. into the mice (3 × 10^6^ cells per mice) 2 days after tumour inoculation. Then, the mice were treated with 250 μg of anti-mouse CD137 mAb (3H3), that was prepared and purified in our laboratory^37^, to activate T cells. In some experiments (presented in Figs 4c and 5b, Supplementary Fig. 9c,e,f; Fig. 7), WT mice were inoculated with CMS5a1 cells at footpads (2 × 10^5^ per foot) and treated with 250 μg of anti-mouse CD137 mAb (3H3) on day 0 and 5, then CD8^+^ T cells were prepared from DL 7 days after the tumour inoculation and used for ACT. In some experiments, some groups of *RAG*^−/−^ mice were treated with ATR inhibitor VE822 (KareBay Biochem. Inc., Monmouth Junction, NJ; 60 mg kg^−1^) on day 5, 7 and 9 (ref. 58). In the experiment using 4T1-HAIFNγTf, 4T1-HAIFNγTf cells, 5 × 10^5^ cells per mouse, were inoculated into *RAG*^−/−^ mice. When palpable tumours developed after 10 days, mice were received DL cells (5 × 10^7^ cell per mice) of WT or *IFN-γ*^−/−^ mice that were inoculated with 4T1-HAIFNγTf cells 7 days earlier. tumour cells were isolated from tumour mass 30 days after ACT.

### ELISA for IFN-γ produced by HA-specific or OVA-specific CTL

HA-specific CTL or CD8^+^ D.L. cells of tumour-bearing WT mice (2.5 × 10^6^ cells per ml) were co-cultured with 4T1 or various 4T1-derived cells (1.25 × 10^6^ cells per ml) for 16 h in 200 μl of RPMI-1640 (Nissui Pharmaceutical, Tokyo, Japan) supplemented with 0.2 mM L-glutamine (Wako), 25 mM NaHCO_3_ (Wako), 10% heat-inactivated fetal calf serum (FCS; JRH biosciences, Lenexa, KA), and 5 × 10^−5^ M β2-mercaptoethanol (Wako) on a 96-well flat-bottomed microtiter plate (Corning, Corning, NY) at 37 °C in a 5% carbon dioxide humidified atmosphere. In the experiments using EG7.1 cells, OVA-specific OT-1 CTL cells were co-cultured with EG7.1 cells. Cell-free culture supernatants were collected, then IFN-**γ** levels were evaluated by using a highly sensitive mouse IFN-**γ** specific enzyme-linked immunosorbent assay (ELISA) kit (Ready-SET-Go!; eBioscience) according to the manufacturer's instructions.

### Cytotoxicity assay

Cytotoxic activity against 4T1 cells and various 4T1 cell-derived clones was evaluated by a standard 4 h ^51^Cr release assay^33^,^59^. Some cells were pre-pulsed with HA peptide (1 μg ml^−1^) for 24 h. Data are represented as the mean±s.d. of triplicate samples.

### Western blotting

Tumour cells were freshly prepared as described above and lysed in RIPA buffer (1% NP40, 50 mM Tris-HCl (pH8.0), 150 mM NaCl, 0.5% deoxycollate and 10% SDS, 1 mM sodium vanadate, 1 mM sodium fluoride, 1 mM phenylmethylsulfonyl fluoride, aprotinin (1 μg ml^−1^) and leupeptin (1 μg ml^−1^)). After passing through a 26 G needle followed by a 30 G needle, total cell lysates were subjected to SDS-polyacrylamide gel electrophoresis (SDS–PAGE) and transferred onto polyvinylidene difluoride membranes (Miliipore). The membranes were analysed with anti-STAT1 mAb (1:1,000 dilution, #9172, Cell Signaling Technology, Beverly, MA), anti-phospho STAT1 (Tyr701) mAb (1:1,000 dilution, #7649, Cell Signaling Technology), anti-phospho STAT1 (Ser727) mAb (1:1,000 dilution, #8826, Cell Signaling Technology), anti-STAT3 (1:1,000 dilution, #9139, Cell Signaling Technology), anti-phospho STAT3 (Tyr705; 1:1,000 dilution, #9145, Cell Signaling Technology) or anti-β-actin antibody (1:500 dilution, Poly6221, BioLegend). The membranes were developed with SuperSignal West Dura Extended Duration Substrate (Thermo Science) and analysed with an OptimaShot CL-420α image analyzer (Wako, Osaka, Japan). All uncropped blots are shown in Supplementary Fig. 10.

### Preparing for the side population and main population

4T1-HA cells were suspended at 1 × 10^6^ cells per ml in culture medium and stained with 9.0 μg ml^−1^ Hoechest 33342 (Sigma-Aldrich, St. Louis, MO) for 90 min at 37 °C (ref. 60). After washing, cells were analysed and sorted by a triple laser MoFlo (Cytomation, Fort Collins, CO) with Summit software (Cytomation) at Keio GCOE FCM Core Facility (Keio University School of Medicine, Tokyo, Japan). Hoechst 33342 was excited at 350 nm, and fluorescence emission was detected by using a 405/BP30 and 570/BP20 optical filter for Hoechst blue and Hoechst red, respectively, and a 550 nm long-pass dichroic mirror (all Omega Optical Inc.) to separate the emission wavelengths. Both Hoechst blue and red fluorescence are shown on a linear scale. Propidium iodide (PI) fluorescence was measured through 630BP30 after excitation at 488 nm with an argon laser, and a live cell gate was defined that excluded the cells positive for PI. Addition of 15 μg ml^−1^ reserpine resulted in the complete disappearance of the side population (SP) fraction (Sigma-Aldrich). Isolated SP and main population (MP) were re-suspended in culture medium and cell number and viability were confirmed. Then, cells were diluted to appropriate injection doses, mixed with BD Matrigel (BD Bioscience) according to manufacturer^61^.

### Array-based comparative genome hybridization analysis

Agilent SurePrint G3 Mouse Microarray 4 × 180 K array technology (Agilent Technology, Inc., Palo Alto, USA) was used to analyse genomic structural variants^62^. Genomic DNA was isolated from tumour cells by chloroform/phenol extraction followed by ethanol precipitation (Sigma). Test and reference genomic DNAs (500 ng per sample) were fluorescently labelled with Cy5 (test samples) and Cy3 (reference: original cells that inoculated into the mice) with a Genomic DNA Enzymatic Labeling Kit (Agilent Technologies). All array hybridizations were performed according to the manufacturer's methods, immediately scanned with a G2565BA Microarray Scanner System (Agilent), and processed by Feature Extraction Software Ver. 10.7.3.1 (Agilent). All regions of statistically significant copy-number change were determined using Aberration Detection Method-2 (ADM2) algorithms on Agilent Genomic Workbench software version 6.5 Lite software (Agilent Technology)^63^. The ADM2 algorithms identify genomic regions with copy-number differences between the test and the reference based on log2 ratios of fluorescent signals from probes in the interval. Results were analysed under conditions that fuzzy zero was ON and Moving Average was set at 60 pt.

### FISH analysis

Metaphase chromosome spreads were prepared from cultured mouse cells using conventional acetic acid-methanol fixation methods. Two bacterial artificial chromosomes (BACs) RP23-357M5 and RP23-146E14 were used to generate region-specific FISH probes for the amplified region (3A1) and for the reference region (3A3), respectively. BAC DNAs were labelled by nick-translation kit (Roche) according to the manufacturer's protocol with Cy5-dUTP (357M5) (Roche) and Green-dUTP (146E14; Abbott). To examine the transduced HA gene, MSCV-HA-IRES-GFP vector was labelled with Cy3-dUTP (Roche) and specific FISH probes for the centromere and telomere of chromosome 17 were labelled with Cy5-dUTP (Roche). The labelled probes were mixed with sonicated salmon sperm DNA and Cot-1 DNA in hybridization solution. The probes were applied to the pretreated sections, covered with coverslips and simultaneously denatured at 70 °C for 5 min. Hybridization was carried out at 37 °C overnight. Slides were then washed with 50% formamide /2 × SSC at 37°C for 20 min, 1 × SSC for 15 min at room temperature, counter-stained by 4,6-diamidino-2phenylindole (DAPI) and mounted. The FISH images were captured with the CW4000 FISH application program (Leica Microsystems Imaging Solution Ltd., Wetzlar, Germany) using a cooled CCD camera mounted on a Leica DMRA2 microscope.

### Quantitative RT–PCR

Total RNA was isolated from freshly prepared tumour cells using RNA STAT-60 (TEL-TEST Inc, Friendswood, TX) and first-stranded cDNA was prepared using oligo dT primers and TaqMan RT Reagents (Applied Biosystems, Foster City, CA). Quantitative PCR was performed following manufacturer's instructions^35^. Briefly, 7500 Real-Time PCR System (Applied Biosystems, Foster City, CA) was used with Assays-on Demand gene expression products (Applied Biosystems) of mouse target genes, *Apobec1* (Mm01184109_m1), *Apobec2* (Mm00477588_m1), *Apobec3* (Mm01298575_m1), *Apobec4* (Mm01287498_m1), *Atm* (Mm01177457_m1), *Atr* (Mm01223626_m1) or endogenous control *GAPDH* (Ma99999915_g1) and TaqMan Universal PCR Master Mix (Applied Biosystems). The expression levels of respective molecules were shown as a ratio compared with GAPDH in the same sample by calculation of cycle threshold (Ct) value in amplification plots with 7500 SDS software (Applied Biosystems). Relative expression levels of respective molecules were calculated by relative quantification (ΔΔCt) using SDS v1.2 with RQ software (Applied Biosystems) according to manufacturer's instructions. Results of all tested individual tumour cell and mean±s.d. are presented. For RT^2^ Profiler PCR Array for Mouse DNA repair (Qiagen, Venlo, Netherlands), cDNA was synthesized from 100 ng of the total RNA using the RT^2^ preAMP cDNA synthesis kit (Qiagen), and the quality of isolated RNA was evaluated using RT^2^ RNA QC PCR Arrays (Qiagen) according to the manufacturer's instructions. After all control tests, the samples were analysed using the RT^2^ Profiler PCR Array performed in 96-well plates on StepOnePlus (Applied Biosystems). The thresholds and baselines were set according to the manufacturer's instructions, and the data were analysed using software supplied on Qiagen homepage on website.

### RT–PCR and genomic PCR

Total RNA was isolated as described above and first-stranded cDNA was prepared using oligo dT primers and TaqManRT Reagents (Applied Biosystems, Foster City, CA). Genomic DNA was prepared using Quiagen DNeay Blood & Tissue Kit (Venlo, Netherlands). PCR reaction was performed^64^ using the following primers: HA33-1026: 5′-GACGGATCCATGAAGGCAAACCTACTGGTC-3′ and 5′-TGATTAACCATCCTCAATTTGGCAC-3′; HA860-1733: 5′-GAAGAGGCTTTGGGGTCCGGCATCATCACC-3′ and 5′-GACGCGGCCGCTCAGATGCATATTCTGCACTG-3′; OVA432-1125: 5′-GCTCATCAATTCCTGGGTAG-3′ and 5′-GTTGGTTGCGATGTGCTTGA-3′; β-actin; 5′-TACGTAGCCATCCAGGCTGT-3′ and 5′-AGGATGCGGCAGTGGCCAT-3′.

To examine the expression of mERK, PCR reaction was performed using 5′-TTGGCATCAATGACAT-3′ and 5′-TGTGGCTACGTACTCTGTC-3′, then PCR products (320 bp of wild-type ERK2 or mERK2 cDNA) were digested by *Sfcl* restriction enzyme (New England Biolabs, Beverly, MA) that selectively cleaves mERK, but not WT ERK2, to generate 159 and 161 bp fragments^29^.

To confirm the genomic alteration of X chromosome of CMS5a1 demonstrated by a-CGH assay, quantitative genomic PCR was performed with Assays-on Demand gene expression products (Applied Biosystems) for a single exon of mouse target genes, *Gm14374* (Mm03059176_gH) located on X A1.1, *Rnf113a1*(Mm02343059_s1) located on X A3.3, or endogenous control *β-actin* (Mm00607939_m1) and TaqMan Universal PCR Master Mix (Applied Biosystems) as described above. Results of all tested individual tumour cell and mean±s.d. are presented.

### Histological examination for phospho-histone H2A.X

CMS5a1 and CMS5a1**γ**RDN cells were inoculated in the left flank and the right flank, respectively, of the same *RAG*^−/−^ mouse. Eighteen days after tumour inoculation, the mice were treated with CD8^+^ DL cells of CMS5a1-bearing WT mice treated with anti-CD137 mAb on the same day and 7 days after tumour inoculation. Tumour masses were harvested on day 20 (2 days after ACT) from ACT-treated mice and control ACT-non-treated mice, fixed in 10% formalin, and then embedded in paraffin. Following hematoxylin/eosin (HE) staining of paraffin sections^65^, to detect of phospho-Histone H2A.X (Ser139), paraffin sections were incubated with rabbit anti-phospho-Histone H2A.X (Ser139)(γH2A.X) mAb (clone 20E3) (Cell Signaling Technology, Denver, MA) and biotin-conjugated goat anti-rabbit IgG (Dako, Carpenteria, CA) in an automated immunostainer (BenchMark; Ventana Medical Systems, Tucson, AZ) by using an iVIEW DAB Detection Kit (Open Secondary; Ventana) and a Cell Conditioning Solution (CC1; Ventana). Finally, sections were counter-stained with hematoxylin, and were scanned in a Virtual Slide System (VS110; Olympus, Tokyo, Japan). The whole area of tumour margin was examined in each specimen, and the numbers of positively nucleus, % of intact area and % of cell death area were calculated by image analysis software, Tissue Studio v.2.3 (Definiens AG, Munich, Germany).

### Statistical analysis

Statistical analysis was performed by unpaired, two-tailed Student's *t*-test for the cytotoxicity and quantitative PCR analysis. *P* values <0.05 were considered as significant.

### Data availability

The array CGH data have been deposited in the NCBI database under the accession code GSE92271. The authors declare that all the other data supporting the findings of this study are available within the article and its Supplementary Information files and from the corresponding author upon reasonable request.

## Additional information

**How to cite this article:** Takeda, K. *et al*. IFN-γ is required for cytotoxic T cell-dependent cancer genome immunoediting. *Nat. Commun.* **8,** 14607 doi: 10.1038/ncomms14607 (2017).

**Publisher's note:** Springer Nature remains neutral with regard to jurisdictional claims in published maps and institutional affiliations.

## Supplementary Material

Supplementary Information Supplementary Figures and Supplementary Table
