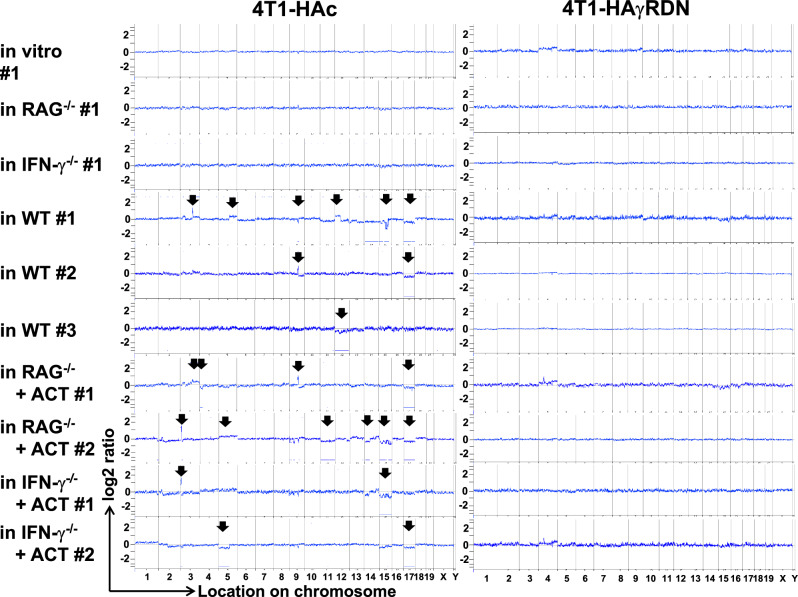# Author Correction: IFN-γ is required for cytotoxic T cell-dependent cancer genome immunoediting

**DOI:** 10.1038/s41467-024-53456-2

**Published:** 2024-12-04

**Authors:** Kazuyoshi Takeda, Masafumi Nakayama, Yoshihiro Hayakawa, Yuko Kojima, Hiroaki Ikeda, Naoko Imai, Kouetsu Ogasawara, Ko Okumura, David M. Thomas, Mark J. Smyth

**Affiliations:** 1 Division of Cell Biology, Biomedical Research Center, Graduate School of Medicine, Juntendo University, Bunkyo-ku, Tokyo 113-8421 Japan; 2 Department of Biofunctional Micribiota, Graduate School of Medicine, Juntendo University, Bunkyo-ku, Tokyo 113-8421 Japan; 3 Department of Immunology, Juntendo University School of Medicine, Bunkyo-ku, Tokyo 113-8421 Japan; 4 Cancer Immunology Program, Peter MacCallum Cancer Centre, St Andrews Place, East Melbourne, 3002 Victoria Australia; 5 Frontier Research Institute for Interdisciplinary Sciences, Tohoku University, Sendai, 980-8578 Japan; 6 Department of Immunobiology, Institute of Development, Aging, and Cancer, Tohoku University, Sendai, 980-8575 Japan; 7 Division of Pathogenic Biochemistry, Department of Bioscience, Institute of Natural Medicine, University of Toyama, Sugitani 2630, Toyama, 930-0194 Japan; 8 Laboratory of Morphology and Image Analysis, Biomedical Research Center, Juntendo University Graduate School of Medicine, Tokyo, 113-8421 Japan; 9 Department of Immuno-Gene Therapy, Mie University Graduate School of Medicine, 2-174 Edobashi, Tsu, Mie 514-8507 Japan; 10 Department of Oncology, Nagasaki University Graduate School of Biomedical Science, 1-12-4 Sakamoto, Nagasaki, 852-8523 Japan; 11 Department of Hematology and Oncology, Icahn School of Medicine at Mount Sinai, 1470 Madison Avenue, New York, New York 10029 USA; 12 Atopy (Allergy) Research Center, Graduate School of Medicine, Juntendo University, Bunkyo-ku, Tokyo 113-8421 Japan; 13 Cancer Division, Garvan Institute of Medical Research, Darlinghurst, New South Wales 2010 Australia; 14 Immunology in Cancer and Infection Laboratory, QIMR Berghofer Medical Research Institute, Herston, 4006 Queensland Australia; 15 School of Medicine, University of Queensland, Herston, 4006 Queensland Australia

Correction to: *Nature Communications* 10.1038/ncomms14607, published online 24 February 2017

In this article, an incorrect panel was inadvertently placed in Fig. 3a. The right bottommost panel should display the result of array-based comparative genome hybridization (array CGH) analysis of 4T1 mammary tumor cells that express influenza haemagglutinin (HA) antigen and a dominant-negative form (DN) of the IFN-γ receptor (IFN-γR) (4T1-HAγRDN) following a one-month subcutaneous growth in IFN-γ deficient mice received with adoptive wild type CTL transfer (ACT) #2 (4T1-HAγRDN in IFN-γ-/- + ACT #2). However, this line figure appears incorrect. Authors have confirmed that all the original source data of the array CGH were correctly deposited in the NCBI database. Accordingly, they have amended the line figure of 4T1-HAγRDN in IFN-γ-/- + ACT #2 with the correct result (raw data file: Fig3a B10-B10_BC.txt in the NCBI database). This panel does not show any significant genomic alteration (DNA copy-number alterations (CNAs)), thus, this correction does not affect the original conclusions. The correct Fig. 3a is shown below: