# Supplementary material for: IFN-γ is required for cytotoxic T cell-dependent cancer genome immunoediting
Source: Nat Commun. 2017 Feb 24;8:14607. doi: 10.1038/ncomms14607 (PMC5333095; doi:10.1038/ncomms14607)
Supplement: Supplementary Information — Supplementary Figures and Supplementary Table [file ncomms14607-s1.pdf]

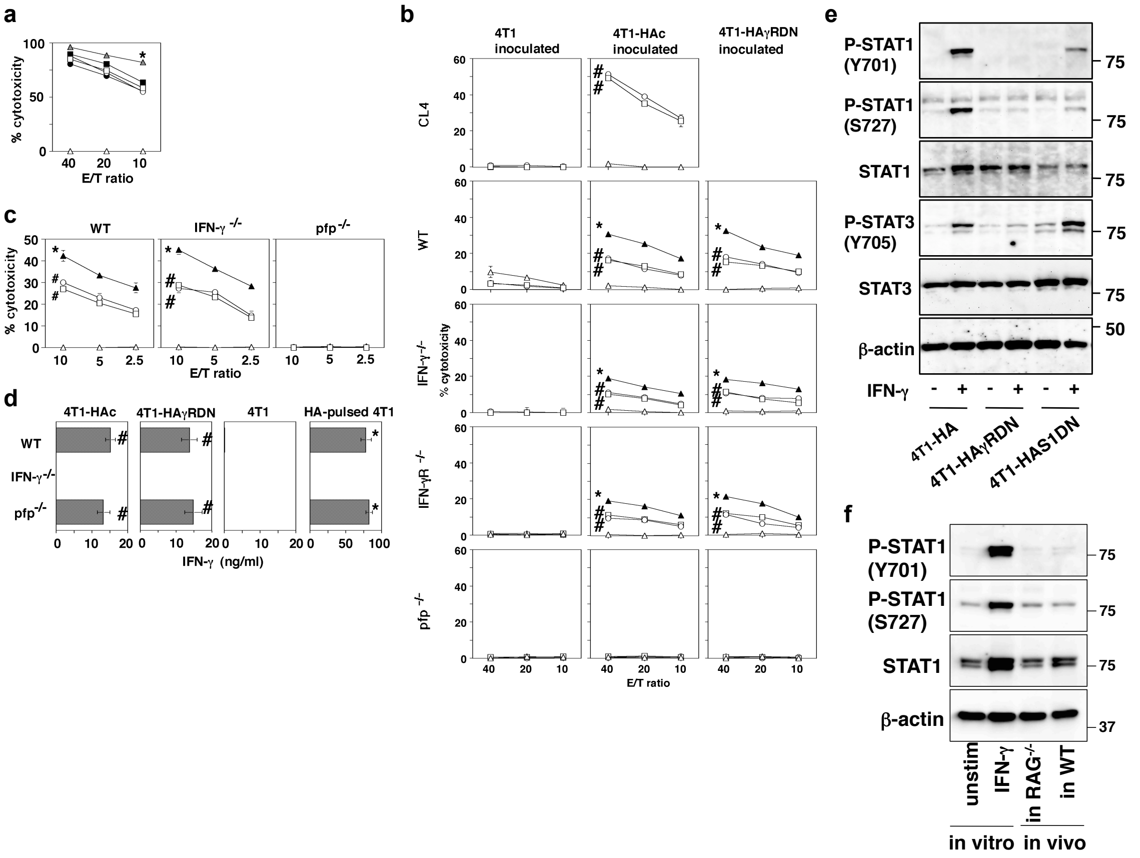

Supplementary Figure 1. HA antigenicity of 4T1-HAc and 4T1-HAγRDN cells. a, 4T1 (triangle), 4T1-HAc (square), and 4T1-HAγRDN (circle) cells were pre-cultured with (black) or without (white) IFN-γ for 24 h, and used as the target cells for cytotoxic analysis of HA-specific WT CTL prepared as described in Material and Methods. 4T1 cells pre-pulsed with HA peptide for 24 h (grey triangle) were also used as the target cells *; $p < 0.05$ as compared with all other cells at the same indicated E/T ratio by unpaired, two-tailed Student's *t* test. Similar results were obtained in three experiments. **b**, 4T1, 4T1-HAc, or 4T1-HAγRDN cells were inoculated into footpads of indicated mice, and $CD8^+$ cells were prepared from popliteal lymph nodes 10 days later. Cytotoxic activity against 4T1 (open triangle), 4T1-HAc (square), and 4T1-HAγRDN (circle) cells was examined. HA-pulsed 4T1 (closed triangle) were also used as the target cell in the experiments using WT, *IFN-γ*$^{-/-}$, *IFN-γR*$^{-/-}$, and *pfp*$^{-/-}$ mice. *; $p < 0.05$ as compared with 4T1, 4T1-HAc and 4T1-HAγRDN cells at all E/T ratio. #; $p < 0.05$ as compared with 4T1 cells at all E/T ratio. Both are analyzed by unpaired, two-tailed Student's *t* test. Similar results were obtained in three experiments. **c** and **d**, 4T1-HA cells were i.p. inoculated into WT, *IFN-γ*$^{-/-}$, or *pfp*$^{-/-}$ mice, and HA-specific CTL were prepared from these mice as described in the Material and Methods. IFN-γ was supplemented during the *in vitro* culture when HA-specific CTL were prepared from *IFN-γ*$^{-/-}$ mice. Then, $CD8^+$ cells were purified and cytotoxic activity against 4T1 (open triangle), HA-pulsed 4T1 (closed triangle), 4T1-HAc (square), and 4T1-HAγRDN (circle) cells was examined. *; $p < 0.05$ as compared with 4T1, 4T1-HAc and 4T1-HAγRDN cells at all E/T ratio. #; $p < 0.05$ as compared with 4T1 cells at all E/T ratio. Both are analyzed by unpaired, two-tailed Student's *t* test. Similar results were obtained in three experiments (**c**). Prepared $CD8^+$ cells were also incubated with 4T1HAc, 4T1-HAγRDN, 4T1, or HA-pulsed 4T1 for 24 h, then IFN-γ levels in the cell-free culture supernatants were determined by ELISA. *; $p < 0.05$ as compared with 4T1, 4T1-HAc and 4T1-HAγRDN cells. #; $p < 0.05$ as compared with 4T1 cells. Both are analyzed by unpaired, two-tailed Student's *t* test. Similar results were obtained in three experiments (**d**). **e**, Cell lysates were prepared from 4T1-HAc, 4T1-HAγRDN and 4T1-HAS1DN cells maintained *in vitro* and those cultured with IFN-γ for 24 h before cell lysate preparation. Phosphorylation of STAT1 and STAT3 in tumor cells was analysed by Western blotting. **f**, Cell lysates were prepared from parental 4T1 cells maintained *in vitro*, those cultured with IFN-γ for 24 h before cell lysate preparation and those grown in *RAG*$^{-/-}$ or WT mice for 15 days. Phosphorylation of STAT1 and STAT3 in tumor cells was analysed by Western blotting. Results are shown as mean ± SD of three independently prepared cells (a-d).

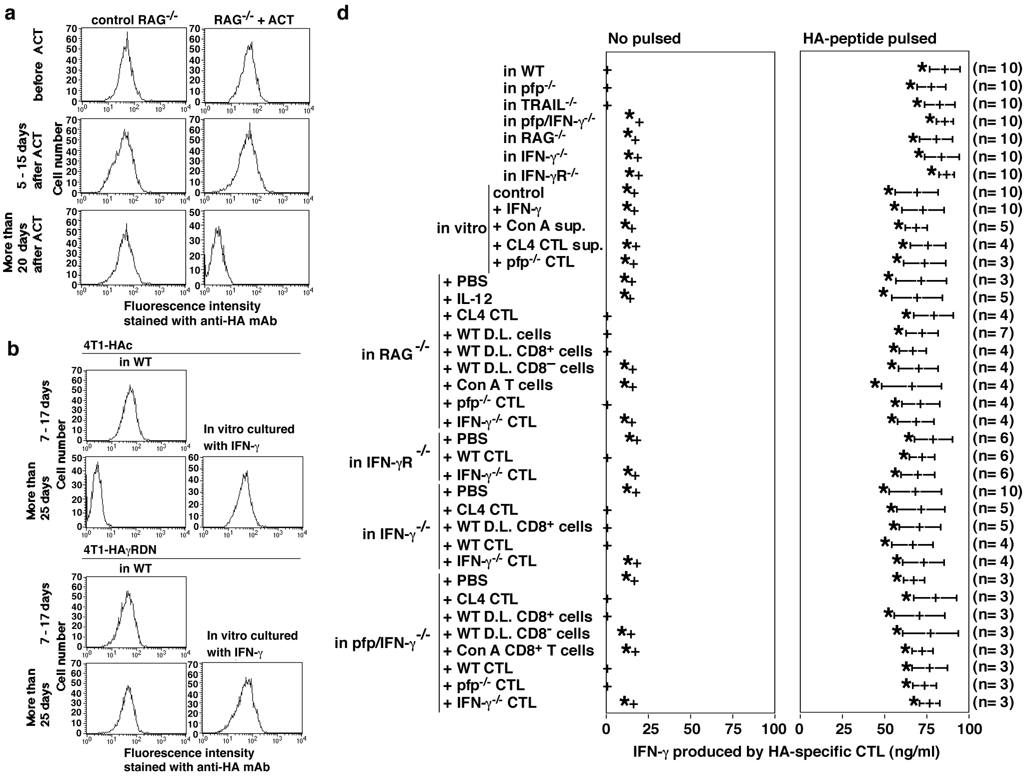

c

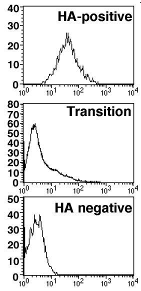

	WT			pfp-/-	TRAIL-/-	pfp/IFN-γ-/-	RAG-/- (no treated)	IFN-γ-/- (no treated)	IFN-γR-/- (no treated)	RAG-/- (WT CTL-treated)	IFN-γ-/- (WT CTL-treated)	IFN-γR-/- (WT CTL-treated)
	15 -18 days	20 - 23 days	> 25 days	> 25 days			> 25 days					
HA-positive	19/24 (79.2%)	2/25 (8.0%)	0/50 (0%)	0/23 (0%)	0/22 (0%)	11/11 (100%)	50/50 (100%)	50/50 (100%)	39/42 (92.9%)	0/20 (0%)	0/25 (0%)	0/20 (0%)
Transition	5/24 (20.8%)	6/25 (24.0%)	4/50 (8.0%)	3/23 (13.0%)	2/22 (9.1%)	0/11 (0%)	0/50 (0%)	0/50 (0%)	3/42 (7.1%)	1/20 (5.5%)	2/25 (8.0%)	1/20 (5.0%)
HA negative	0/24 (0%)	17/25 (68.0%)	46/50 (92.0%)	20/23 (87.0%)	20/22 (90.9%)	0/11 (0%)	0/50 (0%)	0/50 (0%)	0/42 (0%)	19/20 (95.0%)	23/25 (92.0%)	19/20 (95.0%)

Supplementary Figure 2. HA expression and immunogenicity of 4T1-HA and 4T1-HAγRDN cells after growth under several conditions. a, 4T1-HA cells were inoculated into *RAG-/-* mice, and 10 days later some mice were treated with HA-specific WT CTL. 4T1-HA cells were isolated from tumor mass during the indicated period, and HA expression was examined by flow cytometry. Similar results were obtained in three experiments. **b**, 4T1-HAc and 4T1-HAγRDN cells were respectively inoculated into the left flank and the right flank of the same WT mouse, and tumor cells were isolated from tumor mass during the indicated days after tumor inoculation. HA expression on isolated 4T1-HA or 4T1-HAγRDN cells was examined by flow cytometry. HA expression on 4T1-HA cells cultured with IFN-γ for more than 25 days was also examined (right panels). Similar results were obtained in three experiments. **c**, 4T1-HA or 4T1-HAc cells were inoculated into the indicated mice, and tumor cells were isolated during the indicated days after tumor inoculation. Some mice were treated with ACT with HA-specific WT CTL on the same day as tumor inoculation. HA expression on 4T1-HA or 4T1-HAc cells was examined by flow cytometry. The histograms showing HA expression are categorized into three types (positive, transition and negative) and the representative histograms are shown in the left. The results of 4T1-HA and 4T1-HAc were identical, thus we combined these results and present the numbers of experiments showing representative histograms per number of all experiments and the percentages of these in the parentheses. **d**, HA-specific WT CTL were co-cultured for 24 h with following tumor cells that were pre-incubated with or without HA peptide for 24 h; representative HA-negative 4T1-HA cells isolated from WT, *pfp-/-*, or *TRAIL-/-* mice, representative HA-positive 4T1-HA cells isolated from *pfp/IFN-γ-/-*, *RAG-/-*, *IFN-γ-/-*, *IFN-γR-/-* mice, 4T1-HA cells cultured under the indicated condition more than 30 days *in vitro*, 4T1-HA cells isolated from tumor mass in the indicated mice more than 30 days after tumor inoculation. Some of mice were treated with indicated lymphocytes on day 0 or with IL-12 every 3 days. IFN-γ levels in the cell-free culture supernatants were determined by ELISA. The results are shown as the average ± SD of the all tested tumor cells (the number of tests are indicated in parentheses). *, $p < 0.05$ compared with the supernatant harvested from the culture of HA-specific WT CTL with HA no-pulsed HA-negative 4T1-HA cells grown in WT mice by unpaired, two-tailed Student's *t* test.

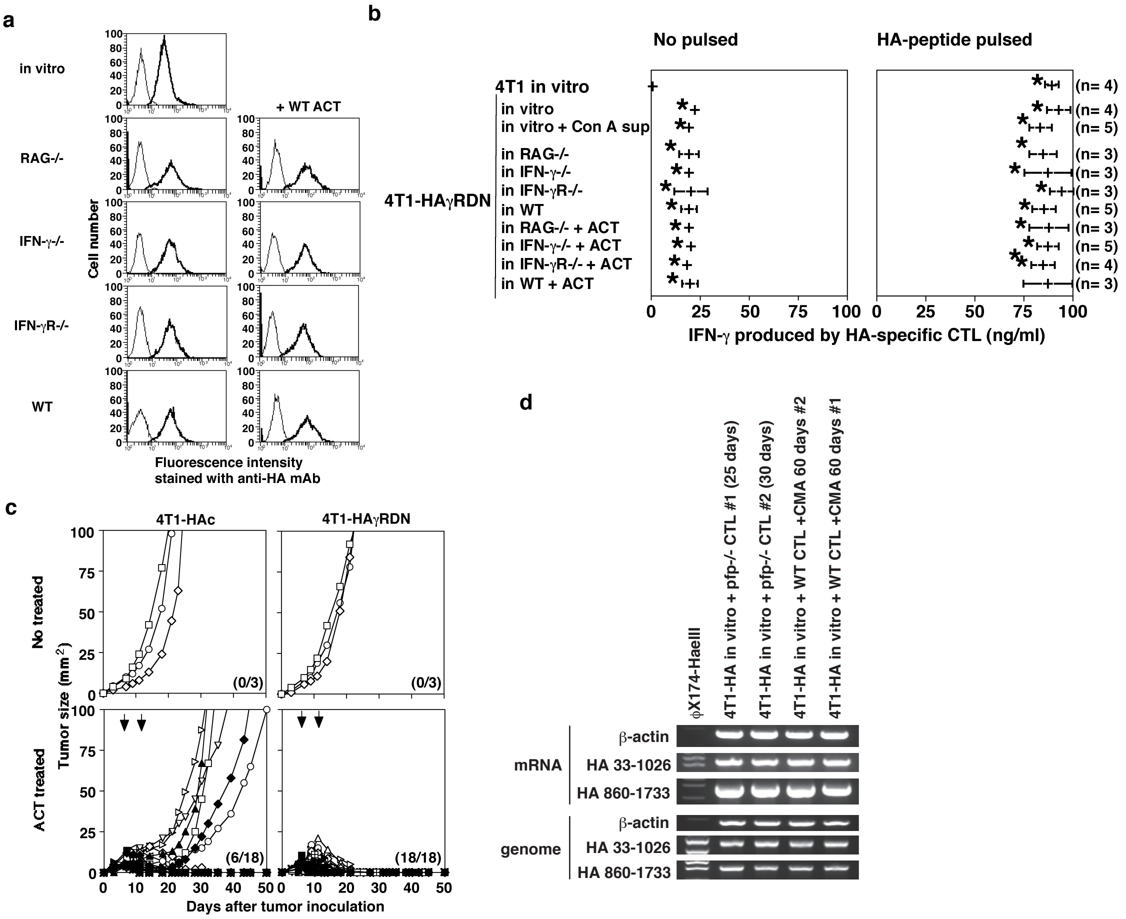

Supplementary Figure 3. HA expression and immunogenicity of 4T1-HAγRDN and 4T10-HA cells after growth under several conditions. a, 4T1-HAγRDN cells were inoculated into the indicated mice and some mice were treated with HA-specific WT CTL. Tumor cells were isolated from tumor masses on day 25, and HA expression was examined by flow cytometry. The thin lines indicate the staining with 2nd antibody (control) and the thick lines indicate the staining with anti-HA mAb followed by 2nd antibody. Similar results were obtained in three experiments. **b**, 4T1-HAγRDN cells were obtained from *in vitro* culture with/without Con A supernatant for 30 days and from tumor masses in indicated mice more than 30 days after tumor inoculation. Some of the mice were treated with WT HA-specific CTL on day 0. HA-specific WT CTLs were co-cultured for 24 h with these 4T1-HAγRDN cells following pre-incubation of these tumor cells with or without HA peptide for 24 h. IFN-γ levels in the cell-free culture supernatants were determined by ELISA. The results are shown as the average ± SD of the all tested tumor cells (the number of tests are indicated in parentheses). *, $p < 0.05$ compared with the supernatant harvested from the culture of HA-specific WT CTL with HA no-pulsed 4T1 cells by unpaired, two-tailed Student's *t* test. **c**,4T1-HAc and 4T1-HAγRDN cells were s.c. inoculated into *RAG*$^{-/-}$ mice. In ACT-treated groups, mice were treated with HA-specific WT CTL on day 7 and 14 as indicated by the arrows (lower panels). Tumor growth was measured, and tumor sizes of individual mice are presented. Tumor rejection rates are indicated in parentheses on every panel. **d,** Genomic DNAs and mRNAs were prepared from 4T1-HA cells cultured with *pfp*$^{-/-}$ HA specific CTL or WT CTL in the presence of 50 nM of perforin inhibitor, concanamycin A (CMA), for the indicated period. Then, indicated segments of the HA gene were amplified by PCR. Representative results are presented from 3 independent experiments.

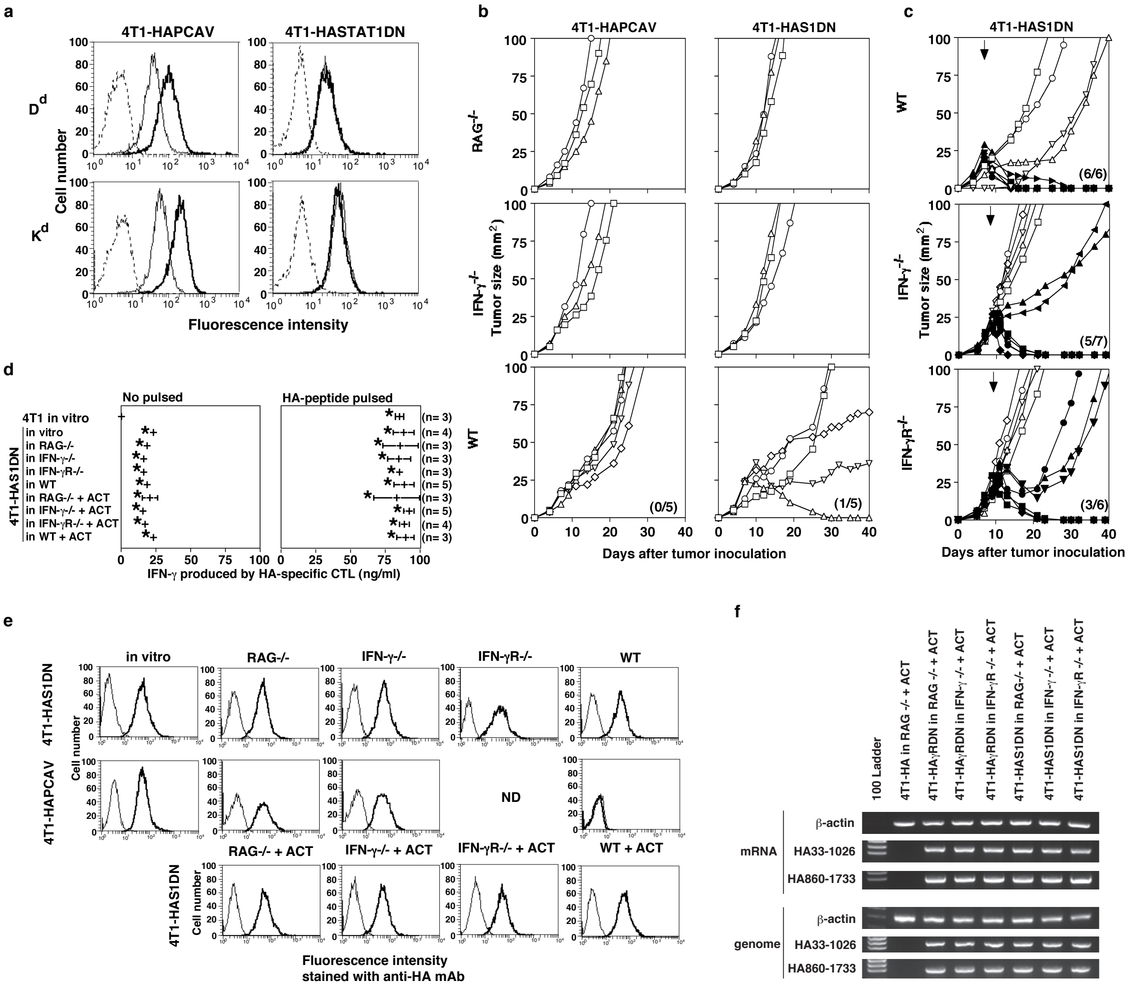

Supplementary Figure 4. HA-specific immunogenicity and HA expression of 4T1-HA cells that express DN STAT1. a, MHC class I expression on 4T1-HAPCAV and DN-STAT1-expressing 4T-HAS1DN cells was analyzed by flow cytometry after 24 h culture with (thick lines), or without (thin line), IFN-γ. Staining of both cell populations with isotype control mAb was indistinguishable after the culture with or without IFN-γ (the level indicated by the dotted line). Similar results were obtained in three experiments. **b,** 4T1-HAPCAV or 4T1-HAS1DN cells were inoculated into the indicated mice, then tumor growth was measured. Tumor rejection rates are indicated in parentheses on the lower panel. Similar results were obtained in three experiments. **c,** 4T1-HAPCAV (open symbols) or 4T1-HAS1DN (closed symbols) cells were inoculated into the indicated mice, then mice were treated with WT HA-specific CTL on day 7 as indicated by the arrows. 4T1-HAPCAV cells were never rejected by ACT on day 7. Tumor rejection rates of 4T1-HAS1DN are indicated in parentheses on every panel. Similar results were obtained in two experiments. **d,** HA-specific WT CTL were co-cultured for 24 h with following 4T1-HAS1DN cells that were pre-incubated with or without HA peptide for 24 h; representative 4T1-HAS1DN cells isolated from *RAG*$^{-/-}$, *IFN-γ*$^{-/-}$, *IFN-γR*$^{-/-}$ or WT mice 25 - 30 days after the tumor inoculation, some of these mice were treated with or without WT HA-specific CTL on day 0, 4T1-HAS1DN cells cultured more than 30 days *in vitro*. IFN-γ levels in the cell-free culture supernatants were determined by ELISA. The results are shown as the average ± SD of the all tested tumor cells (the number of tests are indicated in parentheses). *, $p < 0.05$ compared with the supernatant harvested from the culture of HA-specific WT CTL with HA no-pulsed 4T1 cells by unpaired, two-tailed Student's *t* test. **e**, 4T1HAS1DN or 4T1-HAPCAV cells were inoculated into the indicated mice, and tumor cells were isolated from the growing tumor masses 30 days after tumor inoculation. Some mice were treated with ACT with HA-specific WT CTL on the same day as tumor inoculation. HA expression on isolated tumor cells was examined by flow cytometry. The thin lines indicate the staining with 2nd antibody (control) and the thick lines indicate the staining with anti-HA mAb followed by 2nd antibody. Similar results were obtained in three experiments. **f,** Genomic DNA and mRNAs were prepared from 4T1-HAγRDN or 4T1-HAS1DN cells grown in ACT-treated *RAG*$^{-/-}$, *IFN-γ*$^{-/-}$ or *IFN-γR*$^{-/-}$ mice and 4T1-HA cells grown in ACT-treated *RAG*$^{-/-}$ mice. Then indicated segments of the HA gene were amplified by PCR. Representative results are presented from 3 independent experiments.

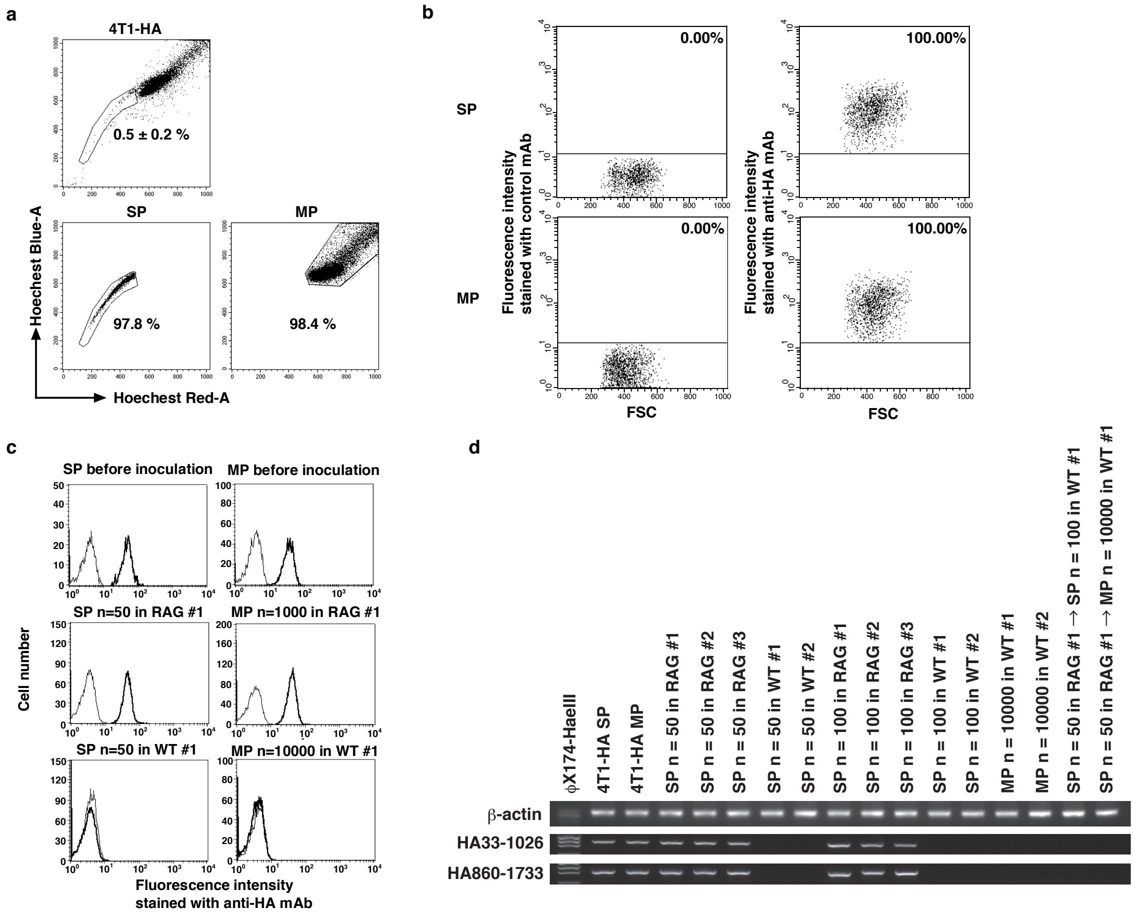

Supplementary Figure 5. HA gene loss in tumor cells derived from SP or MP of 4T1-HA cells in WT mice. a, 4T1-HA cells were stained with Hoechst 33342, and SP and MP were sorted according to the fluorescence emission of Hoechst blue and Hoechst red by a triple laser MoFlo. Addition of 15 μg/ml reserpine resulted in the complete disappearance of the SP fraction. Similar results were obtained in three experiments. **b**, HA expression on freshly isolated 2,000 cells of SP or MP of 4T1-HA cells was examined by flow cytometry. Similar results were obtained in three experiments. **c**, SP or MP of 4T1-HA cells were sorted and indicated number of them were inoculated into the indicated mice, and tumor cells were prepared from tumor mass when tumor grew to 8 mm diameter. HA expression was examined by flow cytometry. Staining with isotype control mAb is indicated by the thin line, and that with anti-HA mAb is indicated by the thick line. Similar results were obtained in two experiments. **d,** Genome DNA was prepared from 4T1-HA cells grown in indicated mice at 8 mm diameter, and the indicated segments of the HA gene were amplified by genomic PCR.

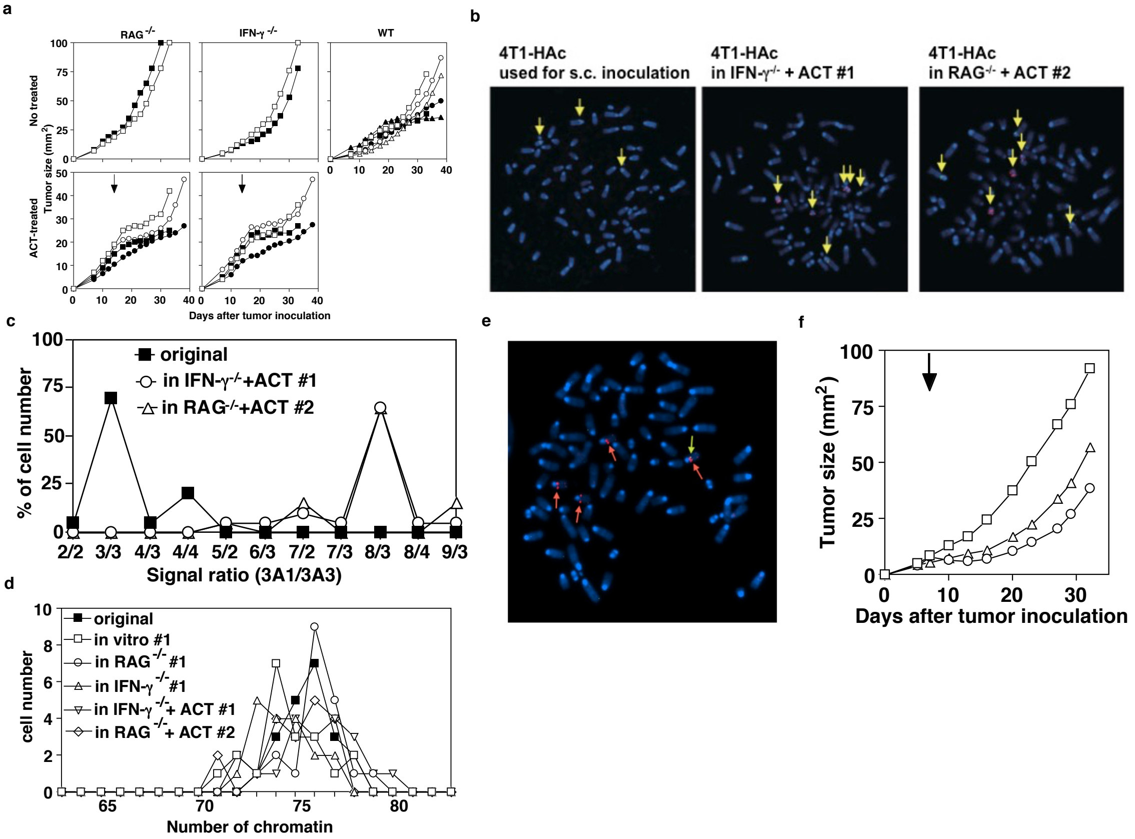

Supplementary Figure 6. FISH analysis of 4T1-HA cells a, Growth of 4T1-HAc and 4T1-HAγRDN cells presented in Fig. 3a. 4T1-HAc cells (open symbols) and 4T1-HAγRDN cells (closed symbols) were respectively inoculated into the left flank and the right flank of the same mouse. Square symbols are indicated as #1, circle symbols are indicated as #2 and triangle symbols are indicated as #3 of respective mice group in Fig. 3a. Tumor-bearing mice were treated with HA-specific WT CTL on day 14 (indicated by the arrows) in ACT-treated groups (in lower panels). Tumor growth was measured and tumor cells were isolated 33 – 38 days after tumor inoculation. **b** and **c**, FISH analysis for 3A1 region in 4T1-HAc cells. Metaphase chromosomes of 4T1-HAc cells that were used for s.c. inoculation (original) and grown in ACT-treated *IFN-γ*$^{-/-}$ mice #1 in Figure 3a were examined by FISH analysis using fluorescence-labeled BAC DNA clone for 3A1(Cy5-dUTP: red). BAC DNA probe for 3A3 (RP23-146E1) was used as the reference (Green-dUTP: green). Representative images are presented in (**b**) and signal ratios (3A1/3A3) are presented in the graph (**c**). Arrows indicate fluorescence signals. **d**, Chromosome number was counted on the FISH images counter-stained by DAPI for 20 cells of indicated 4T1-HAc cells. **e**, FISH analysis for transfected HA gene in 4T1-HAc cells. Metaphase chromosomes of 4T1-HAc cells were examined by FISH analysis using fluorescence-labeled MSCV-HA-IRES-GFP vector (Cy3-dUTP: green) and specific FISH probes for the centromere and telomere of chromosome 17 were labeled with Cy5-dUTP (Cy5-dUTP: red). Representative images are presented. Green arrow indicates MSCV-HA-IRES-GFP vector signal and red allows indicate signal of chromosome 17 specific probes. **f**, Growth of 4T1-HA tumor cells in ACT-treated *pfp/IFN-γ*$^{-/-}$ mice presented in Figure 3b. 4T1-HA tumor cells were inoculated into *pfp/IFN-γ*$^{-/-}$ mice then treated with PBS (square), HA-specific *IFN-γ*$^{-/-}$ CTL #1 (circle) or HA-specific *pfp*$^{-/-}$ CTL #1(triangle) on day 7 as indicated by the arrow, and the subcutaneous growth of these cells was examined. Tumor grew similarly in #1 - #3 mice in respective groups.

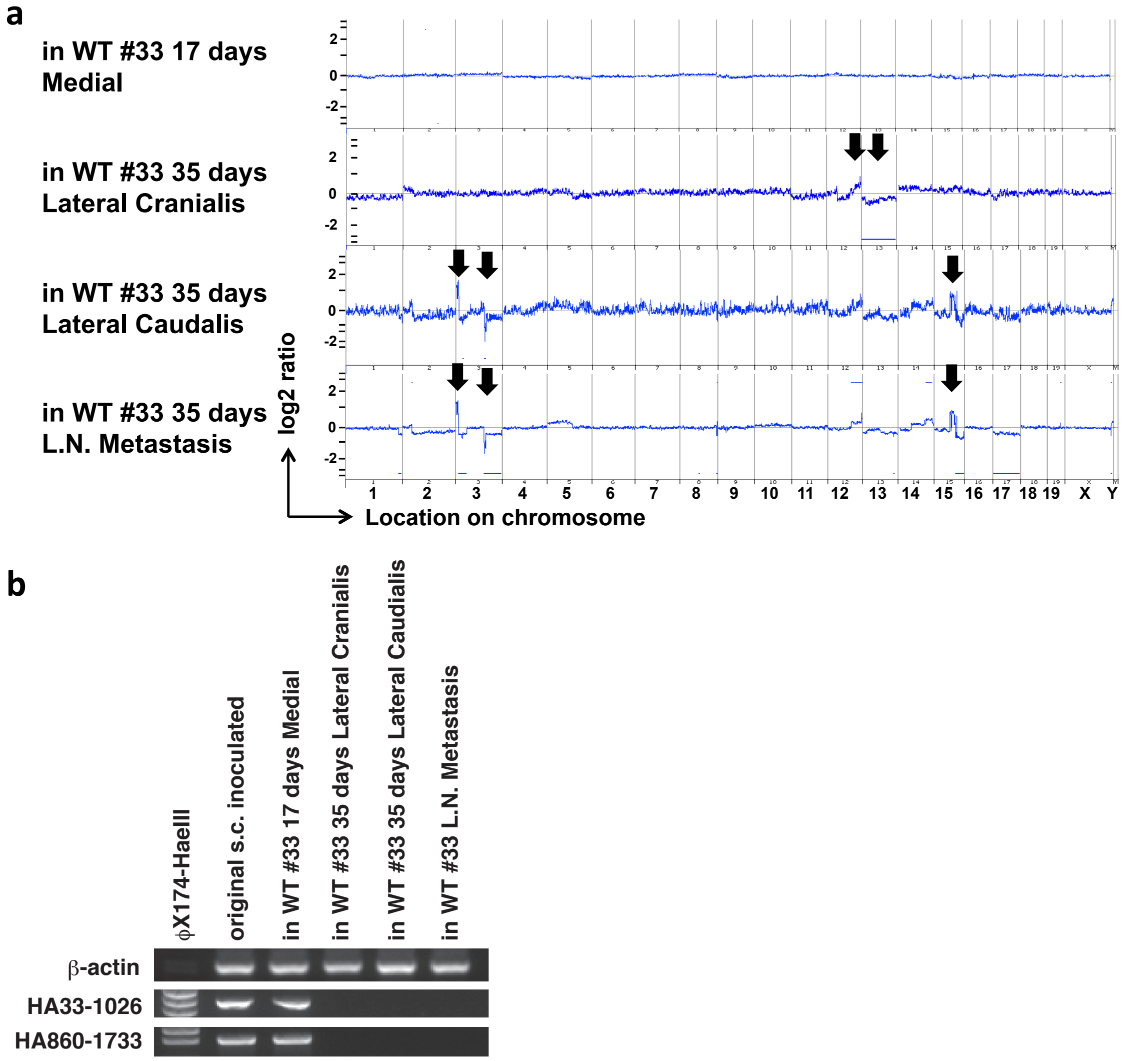

Supplementary Figure 7. Intra-tumor heterogeneity in CNAs of 4T1-HA cells that progressively grow in WT mice. a and **b.** Genomic DNAs were obtained from 4T1-HA cells prepared from the resected medial part 17 days after tumor inoculation and 4T1-HA cells prepared from the cranialis (head) and caudialis (tail) portions of the residual lateral part of the tumor mass 35 days after the tumor inoculation in WT mice. Genomic DNAs were also obtained from metastasized lymph node on day 35 days. Then, CNAs were examined by a-CGH (**a**) and HA gene was examined by genomic PCR of the indicated portions of the HA gene (**b**). In a-CGH analysis, the tumor cells used for the s.c. inoculation are the reference sample, and the positions showing significant CNA are indicated by the lines and arrows.

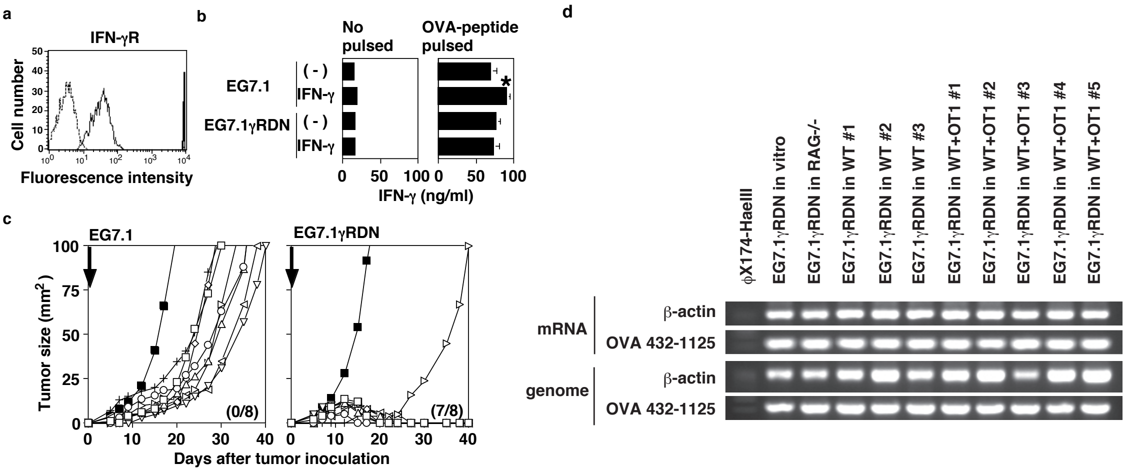

Supplementary Figure 8. Expression of IFN-γR DN attenuated the growth and OVA gene loss of EG7.1 cells *in vivo*. **a**, IFN-γRα chain expression on EG7.1 (thin line) and EG7.1γRDN (thick line) cells were analyzed by flow cytometry. Staining of EG7.1 and EG7.1γRDN cells with isotype control mAb was indistinguishable as indicated by the dotted line. Similar results were obtained in two experiments. **b**, After the incubation with or without OVA peptide in the presence or absence of IFN-γ for 24 h, EG7.1 and EG7.1γRDN cells were co-cultured with OVA-specific OT-1 cells for 24 h, then IFN-γ levels in the cell-free culture supernatants were determined by ELISA. The results are shown as mean ± SD of three independently cultured cells. *; $p < 0.05$ as compared with the supernatant harvested form the culture of the same cells that were pre-incubated without IFN-γ by unpaired, two-tailed Student's *t* test. **c**, EG7.1 and EG7.1γRDN cells were inoculated into $RAG^{-/-}$ mice that were treated with OT-1 cells (5 x 10^6 cells/mice) on day 0 as indicated by the arrow, and the subcutaneous growth of these cells was examined. Closed square symbol in the panels indicates the representative growth of tumor cells in $RAG^{-/-}$ mouse. Tumor rejection rates are indicated in the parentheses on panels. Similar results were obtained in two experiments. **d**, EG7.1γRDN cells were inoculated into $RAG^{-/-}$ or WT mice and some of WT mice were treated with OVA-specific OT1 cells at the same day of tumor inoculation. Genomic DNAs and mRNAs were prepared from the growing tumor mass in the indicated mouse at day 25. Then, indicated segments of the HA gene were amplified by PCR.

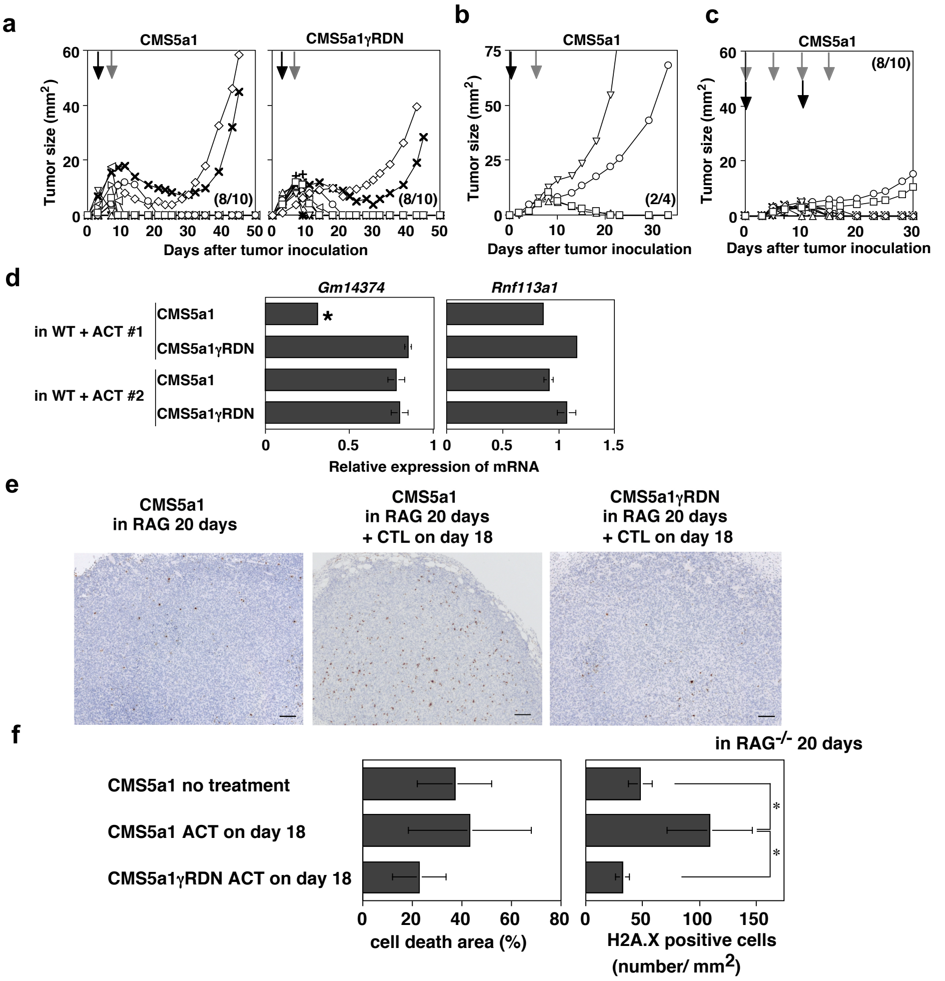

Supplementary Figure 9. Increased foci of phospho-Histone H2A.X (Ser139)(γH2A.X) in CMS5a1 cells grown in *RAG*$^{-/-}$ mice upon ACT a-c, Growth of CMS5a1 and CMS5a1γRDN cells presented in Fig. 5b. CMS5a1 or CMS5a1γRDN cells were inoculated into the same WT mice, then mice were treated with mutated ERK-specific DUC17 T cells 2 days after tumor inoculation as indicated by the black arrow (**a**). CMS5a1 cells were inoculated into WT mice, the mice were treated with CD8 DL cells of CMS5a1-bearing WT mice on the same day as tumor inoculation (**b**) or on the same day and day 10 (**c**) as indicated by the black arrow. The mice were treated with 250 μg of anti-mouse CD137 mAb on day 7 (**a** and **b**) or day 0, 5, 10 and 15 (c) to activate T cells as indicated by grey arrow. Tumor growth was measured and tumor cells were isolated 30 – 50 days after tumor inoculation. Diamond symbols indicate tumor growth in #1 mice and cross symbols indicate those of #2 mice (**a**), circle symbols indicate tumor growth in #3 mice and inverted triangle symbols indicate that in #4 mice (**b**) and square symbols tumor growth in #5 and diamond symbols indicate that in #6 mice (**c**) indicated in Fig. 3 d and e. **d.** Confirmation of copy number reduction in X A1.1 region that was demonstrated by a-CGH array analysis of CMS5a1 cells grown in ACT-treated WT mice #1. Genomic DNAs were prepared from CMS5a1 and CMS5a1γRDN grown in ACT-treated WT mice #1 and #2, and quantitative genomic PCR was performed for a single exon of indicated target genes (*Gm14374* (Mm03059176_gH) located on X A1.1, *Rnf113a1* (Mm02343059_s1) located on X A3.3). The gene expression was normalized to *β-actin* levels, and the relative expression was compared with the respective tumor cells maintained *in vitro*. Results are presented as mean ± SD of 3 experiments. ; $p < 0.05$ as compared with all the other results presented in the same panel by unpaired, two-tailed Student's *t* test. **e** and **f,** Increased γH2A.X positive cells in CMS5a1, but not CMS5a1γRDN, cells grown in ACT-treated *RAG*$^{-/-}$ mice. CMS5a1 and CMS5a1γRDN cells were inoculated in the left flank and the right flank, respectively, of the same *RAG*$^{-/-}$ mouse. Eighteen days after tumor inoculation, the mice were treated with CD8 DL cells of CMS5a1-bearing WT mice treated with anti-CD137 mAb on the same day and 7 days after tumor inoculation. Micrometer cryostat sections of tumor mass were obtained on day 20 (2 days after ACT) from ACT-treated mice and control ACT-non-treated mice. After the staining with hematoxylin/eosin (HE), immunohistochemistry staining was performed with rabbit anti-phospho-Histone H2A.X (Ser139)(γH2A.X) mAb and biotin-conjugated goat anti-rabbit IgG. The whole area of tumor margin in each specimen was scanned in a Virtual Slide System, and the numbers of positive nuclei, and % of cell death area were calculated by image analysis software, Tissue Studio v.2.3. Three to four typical areas of tumor margin were examined in each specimen and the representative photos are presented. Scale bar demonstrates 100 μm (**e**). Results are presented as mean ± SD of 3 tumors (**f**). *; $p < 0.05$ as compared with all the other results presented in the same panel by unpaired, two-tailed Student's *t* test. Similar results were obtained in two independent experiments.

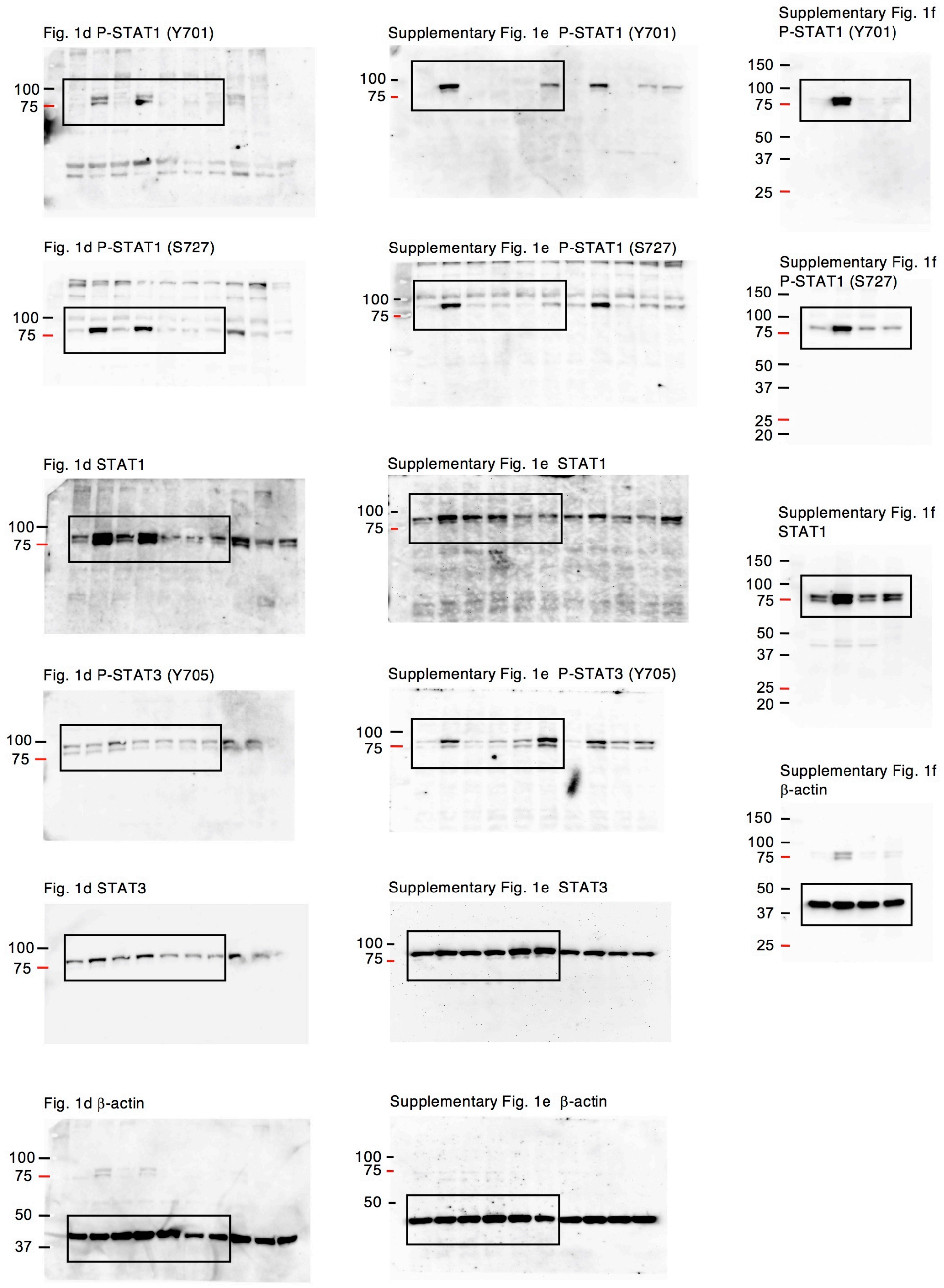

Supplementary Figure 10. Uncropped Western blots. Original uncropped results of scanned Western blotting are presented.

Mice	Cell number	50	100	500	1000	10000	100000
RAG	SP	3/5	5/5	6/6	3/3	ND	ND
	MP	ND	0/10	ND	3/6	6/6	ND
WT	SP	2/10	2/10	1/4	3/3	ND	ND
	MP	ND	ND	ND	0/5	3/6	3/3

number of tumor formed / number of tumor injected

Tumor formation was considered failed when no tumor mass was visible 3 months after tumor inoculation.

ND : not done

Supplementary Table 1. Tumor rejection rate of SP or MP population of 4T1-HA cells.
Indicated number of isolated SP or MP population of 4T1-HA tumor cells was inoculated into the indicated mice, then tumor growth was observed for 3 months.
